# A 3D Searchable Database of Transgenic Zebrafish Gal4 and Cre Lines for Functional Neuroanatomy Studies

**DOI:** 10.3389/fncir.2015.00078

**Published:** 2015-11-24

**Authors:** Gregory D. Marquart, Kathryn M. Tabor, Mary Brown, Jennifer L. Strykowski, Gaurav K. Varshney, Matthew C. LaFave, Thomas Mueller, Shawn M. Burgess, Shin-ichi Higashijima, Harold A. Burgess

**Affiliations:** ^1^Division of Developmental Biology, Eunice Kennedy Shriver National Institute of Child Health and Human Development, National Institutes of HealthBethesda, MD, USA; ^2^Neuroscience and Cognitive Science Program, University of MarylandCollege Park, MD, USA; ^3^Translational and Functional Genomics Branch, National Human Genome Research Institute, National Institutes of HealthBethesda, MD, USA; ^4^Division of Biology, Kansas State UniversityManhattan, KS, USA; ^5^National Institutes of Natural Sciences, Okazaki Institute for Integrative Bioscience, National Institute for Physiological SciencesAichi, Japan

**Keywords:** zebrafish, transgenic, Gal4, Cre, microRNA, 3D registration

## Abstract

Transgenic methods enable the selective manipulation of neurons for functional mapping of neuronal circuits. Using confocal microscopy, we have imaged the cellular-level expression of 109 transgenic lines in live 6 day post fertilization larvae, including 80 Gal4 enhancer trap lines, 9 Cre enhancer trap lines and 20 transgenic lines that express fluorescent proteins in defined gene-specific patterns. Image stacks were acquired at single micron resolution, together with a broadly expressed neural marker, which we used to align enhancer trap reporter patterns into a common 3-dimensional reference space. To facilitate use of this resource, we have written software that enables searching for transgenic lines that label cells within a selectable 3-dimensional region of interest (ROI) or neuroanatomical area. This software also enables the intersectional expression of transgenes to be predicted, a feature which we validated by detecting cells with co-expression of Cre and Gal4. Many of the imaged enhancer trap lines show intrinsic brain-specific expression. However, to increase the utility of lines that also drive expression in non-neuronal tissue we have designed a novel UAS reporter, that suppresses expression in heart, muscle, and skin through the incorporation of microRNA binding sites in a synthetic 3′ untranslated region. Finally, we mapped the site of transgene integration, thus providing molecular identification of the expression pattern for most lines. Cumulatively, this library of enhancer trap lines provides genetic access to 70% of the larval brain and is therefore a powerful and broadly accessible tool for the dissection of neural circuits in larval zebrafish.

## Introduction

A full understanding of how the brain processes sensory information to control behavior requires the cellular-level characterization of the morphology, connectivity, and function of individual neurons. To this end, genetic tools are increasingly used to activate transgene expression in subsets of neurons with a high degree of cell-type specificity. A powerful repertoire of transgenic methods has been developed to visualize, monitor, and manipulate neurons in larval zebrafish. However, the usefulness of these tools depends on the precision with which their expression can be reproducibly driven in small defined populations. In many cases, transgene expression is neither specific to the brain, nor restricted to a sufficiently discrete set of neurons to allow for functional interrogation of the nervous system.

Several strategies have been used for genetically targeting neurons. Promoter fragments, containing cis-regulatory elements from genes expressed in target neurons often allow reporter genes to be expressed in the same cells (Higashijima et al., 1997). Larger genomic regions cloned into bacterial artificial chromosomes have also been successfully used to faithfully recapitulate gene-specific patterns (Jessen et al., 1999; Suster et al., 2009). However, as few genes are expressed in small numbers of neurons, these approaches seldom yield highly discrete domains of reporter expression. Moreover, the generation of each such line is often resource and labor intensive. In contrast, a large number of transgenic “enhancer trap” lines can be rapidly and easily generated through random transposase-mediated transgene integration where transgene expression is directed by regulatory elements flanking the site of integration (Bayer and Campos-Ortega, 1992; Kawakami et al., 2004; Parinov et al., 2004). In particular, the high germ line efficiency of Tol2 has enabled several large scale Gal4 enhancer trap screens (Davison et al., 2007; Scott et al., 2007; Asakawa et al., 2008) and provided transgenic zebrafish lines that have proven powerful tools for both imaging as well as interrogating the function of the nervous system (Scott and Baier, 2009). Still, only a small proportion of these enhancer trap lines show strongly restricted patterns of expression. An alternative strategy was recently tested in which a large number of ~3 kbp genomic DNA fragments from neuronal genes was used to generate transgenic lines (Pfeiffer et al., 2008). It was hypothesized that these fragments, each containing only a subset of gene regulatory elements, would drive highly restricted patterns of neuronal expression. However, from more than 7000 such fragments tested in *Drosophila*, less than 0.1% directed reporter expression to a single neuronal cell type (Jenett et al., 2012). Thus, at present, small groups of neurons—required for functional interrogation—cannot be reliably targeted using single transgene methods alone.

A promising approach is to use intersectional strategies that require two transgenes to be expressed in the same cell to activate expression of a reporter gene. To implement such methods, a number of extensions to the classic Gal4/UAS system have been introduced, including split-Gal4 and expression of the Gal4 inhibitor protein, Gal80 (Pfeiffer et al., 2010; Faucherre and Lopez-Schier, 2011). Recombinases such as Cre have also been used to further restrict Gal4 expression domains, for example by removing a stop cassette downstream of the UAS promoter (Stockinger et al., 2005). Gal4 and Cre driver lines have successfully been used for intersectional approaches in zebrafish (Satou et al., 2013), making this an appealing strategy to achieve highly targeted reporter expression in neuronal cell types.

To expedite the design and maximize the utility of such intersectional strategies, Gal4 and Cre expression patterns must be spatially characterized at high resolution. Only recently have cellular level resolution images of Gal4 enhancer trap lines been made available (Otsuna et al., 2015). Moreover, these expression patterns should ideally be integrated into a common spatial coordinate system in order to predict domains of overlapping expression, enabling researchers to better identify combinations of Gal4 and Cre transgenic lines that label select neurons of interest. This problem can be solved through inclusion of a reference channel and registration of confocal scans to a single common reference brain. Rigorous methods that correct for small inter-individual differences in brain structure or morphology have been developed and applied extensively to human magnetic resonance imaging data. Such brain registration techniques have also been applied to confocal imaging data from *Drosophila* and more recently to larval zebrafish brain scans (Peng et al., 2011; Ronneberger et al., 2012; Portugues et al., 2014; Randlett et al., 2015). These studies illustrate the feasibility and potential utility of applying volumetric registration techniques to confocal imaging data at the scale of the larval zebrafish brain.

Here, we present a database of the expression patterns of 109 transgenic lines that have been registered into a common reference space, derived from 354 high resolution brain scans. We provide “brain browser” software that facilitates the rapid visualization of multiple lines and enables searching lines using anatomic labels, or user defined 3-dimensional regions of interest. The database includes 80 Gal4 enhancer trap lines and 9 Cre enhancer trap lines. To aid in navigating the brain and interpreting enhancer trap expression patterns, we also imaged 20 transgenic lines with genetically defined expression patterns. We show that the browser can be used to find enhancer trap lines that label specific neurons, or even the projection targets of neurons of interest. The browser also enables the intersectional expression domains of Cre and Gal4 lines to be predicted. As a step toward future systematic functional analyses of brain function, the browser also allows a given expression pattern to be used as the template to delineate a set of transgenic lines that cover the same region with minimal overlap.

Transgenic lines with minimal expression outside the brain are the most valuable for neuroscience research. While we have previously shown that non-neuronal expression can be suppressed by inclusion of one or more neuronal restrictive silencing elements in the transgene vector (Bergeron et al., 2012; Xie et al., 2012), this technique cannot be retroactively applied to refine expression of many valuable existing Gal4 lines. Instead, here we tested synthetic 3′ untranslated regions (UTRs) that incorporate target sites for microRNAs that are highly expressed in non-neuronal tissues for their ability to suppress expression outside the brain. MicroRNAs are 21–22 nucleotide ribonucleic acid hairpins that repress translation or destabilize messenger RNA (Bartel, 2009; Mishima et al., 2012; Subtelny et al., 2014). Each microRNA suppresses the expression of target genes that contain 7–8 nucleotides that are complementary to the microRNA “seed” sequence. We show that one synthetic 3′ UTR (utr.zb3), containing target sites for several non-neuronal microRNAs, strongly suppresses expression of a UAS driven reporter in heart, muscle and skin. This synthetic UTR thus enables the use of numerous lines previously inaccessible to desirable experimental manipulations.

## Results

### 3-dimensional spatial registration of Gal4 lines

**Figure 1:**
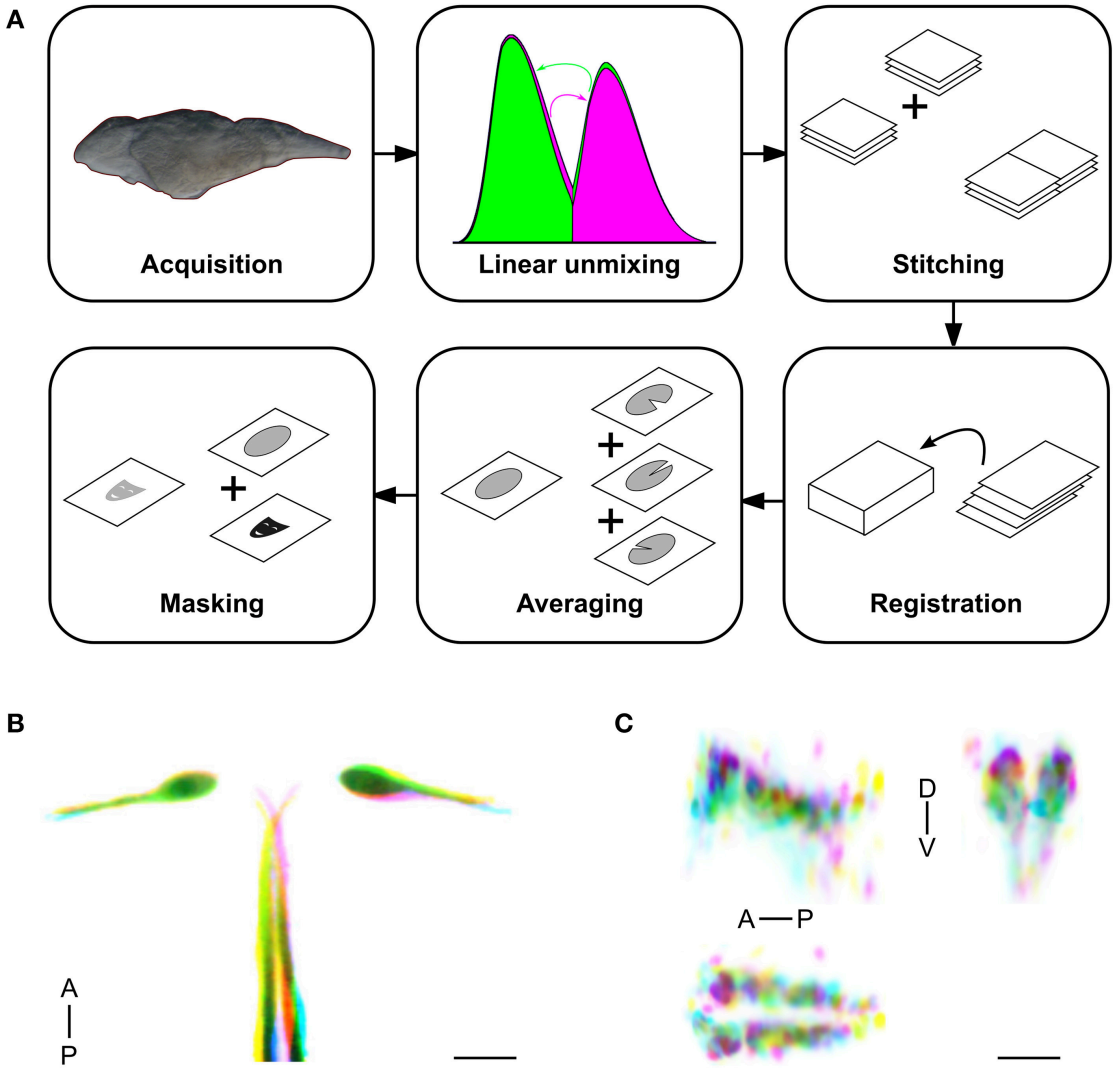
**Method for high throughput, accurate registration of 6 dpf larval brains. (A)** Acquisition and computation pipeline, detailed protocols for each step are described in the Materials and Methods section. **(B)** Overlay of the Mauthner cell, from single image stacks taken from three different *y264Et* larvae (color-coded yellow, pink and blue respectively). Good correspondence is seen for the soma and lateral dendrite region, with more variability in the position of the axon. Scale bar 25 μm. **(C)** Overlay of the superior raphe nucleus, from averaged image stacks for lines *y228Tg* (pink), *y293Et* (blue), and *y308Et* (yellow), shown using a sagittal projection through the region (top left), a transverse projection (top right) and horizontal projection (bottom). Anterior (A), Posterior (P), Dorsal (D), Ventral (V). Scale bar 25 μm.

To construct a high resolution database of enhancer trap lines, we first developed a pipeline for generating average brain representations for transgenic lines (Figure 1A). We used *UAS:Kaede* as a reporter to visualize Gal4 expression, and the *vglut2a:DsRed* transgenic line, which has a broad pattern of DsRed expression, as a reference channel for image registration. At 6 dpf, brain images of live *Gal4, UAS:Kaede, vglut2a:DsRed* larvae were acquired with a confocal microscope at single micron resolution. To reduce scan times, we collected Kaede and DsRed fluorescence simultaneously, with post-scan linear unmixing to eliminate cross-talk between channels. We acquired two image stacks for each brain, covering rostral and caudal regions, which were stitched together prior to image registration. For registration, we used the Computational Morphometry Toolkit (CMTK; Rohlfing and Maurer, 2003). To find optimal registration parameters, we used a calibration set, comprising larvae that were scanned, remounted and rescanned. Using normalized cross-correlation as a measure of registration quality between duplicate scans of the calibration set, we identified an individual *vglut2a:DsRed* brain that yielded the best registrations to use as the reference. We then systematically tested registration parameters to obtain the most accurate alignments while minimizing computation time (see Materials and Methods: Registration and Supplemental Figures 1A–D for additional details).

Based on findings from previous work (Rath et al., 2012), we averaged imaging data from a minimum of three larvae per line to generate representations of Gal4 expression. Averaging helped to fill-in information that was incomplete in any one larva due to variegated expression, yielding a more accurate representation of the expression present in each line than data from a single fish. Next we applied a mask to the average image stack that removed expression data and background fluorescence from outside the brain, enabling 3D visualization of brain expression patterns. To assess the accuracy of our registration pipeline, we compared registered images acquired from transgenic larvae with overlapping expression patterns. *j1229aGt* and *y264Tg* both label the Mauthner cell (Burgess et al., 2009; Tabor et al., 2014). The *vglut2a:DsRed* reference channel does not label these neurons; nevertheless, Mauthner cell bodies were brought into close alignment in three individual *y264Tg* larvae (Figure 1B). Similarly, the Mauthner cells show near perfect overlap between the average representations of *y264Tg* and *j1229aGt* (Supplemental Figures 2A,B). To assess the accuracy of registration for smaller, potentially more variable cell populations, we compared lines *y228Tg, y293Et*, and *y308Et* which all label the superior raphe nucleus as part of their expression domain. Within the raphe, expression patterns registered to within approximately 1 cell diameter (Figure 1C). Next, we compared two average representations for enhancer trap line *y339Et* that were produced from independent sets of larvae. Projections and slices from these stacks were visually similar (Supplemental Figures 2C,C′,D). Finally, we noted that after registration, vascular GFP expression in *flk:GFP* aligned well with gaps in the pan-neuronal *HuC:Cer* pattern, confirming that our imaging pipeline yielded accurate representations of transgene expression patterns (Supplemental Figures 2E,F).

### Characterization of enhancer trap lines by brain imaging and integration mapping

**Figure 2:**
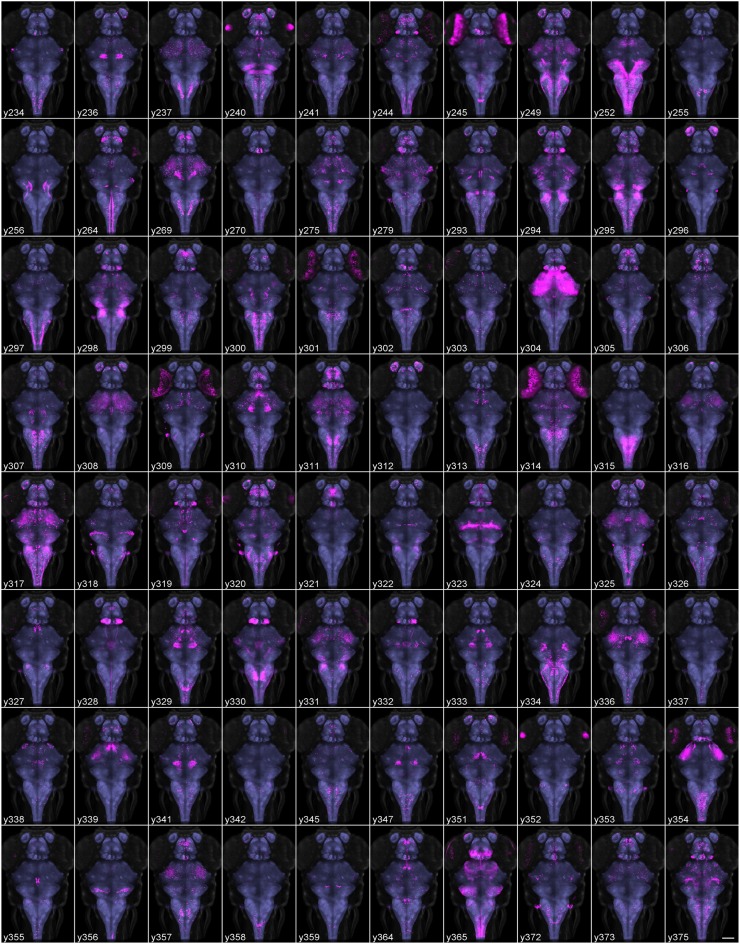
**Average brain representations of Gal4 enhancer trap lines**. Maximum projections through 80 Gal4 enhancer trap lines that were imaged, registered and averaged for this study. Gal4 expression was visualized using the *UAS:Kaede* transgenic line (magenta). Each panel is the average of at least three brains. For contrast, each panel also shows ubiquitous β*actin:Switch* expression (gray) and pan-neuronal *HuC:Cer* expression (blue, after application of brain mask). Scale bar (bottom right panel) 100 μm.

We previously generated more than 200 transgenic Gal4 lines while developing a new vector that allows for brain-specific enhancer trapping (Bergeron et al., 2012). Additionally, during the isolation of transgenic Gal4 lines that used defined promoter elements from the *tph2* and *dbh* genes, we retained several lines with robust Gal4 expression that extended beyond the corresponding gene expression domain, and that are thus effectively enhancer traps. Based on visual inspection of expression patterns under epifluorescence, we selected 80 lines that appeared most useful for anatomical and behavioral experiments, to characterize using high resolution imaging and brain registration (Table 1). In addition, we selected 20 transgenic lines that were generated using BACs or promoters from genes with well-defined expression patterns, thus providing anatomical landmarks and cell type information for annotating the Gal4 enhancer trap lines (Supplemental Figure 3A). We then scanned the brains of 256 Gal4-expressing and 98 other transgenic larvae (Figure 2, Supplemental Figure 3B), comprising 3–10 individuals for each line. Images were processed using the pipeline described above, producing a single representation of each line. We manually annotated neuroanatomical structures labeled by each line, and collected an epifluorescent image to characterize the extent of non-neuronal expression (Supplemental Table 1).

**Table 1:** **Summary of enhancer trap lines imaged**.

**Vector**	**Imaged**	**Neural specific**	**Mapped**	**Integration site**			
				**Intergenic**	**Exon**	**Intron 1**	**Intron 2+**
cfos:Gal4	19	5	11	10	–	–	1
REx2-cfos:Gal4	18	16	12	6	1	2	3
SCP1:Gal4	13	1	8	2	3	–	3
REx2-SCP1:Gal4	24	7	14	6^b^	2	2	4
E-trap:Gal4^a^	6	4	5	3^b^	–	2	–
REx2-SCP1:Cre	6	4	–	–	–	–	–
attp-REx2-SCP1:Cre	3	2	–	–	–	–	–
Total	89	39	50	27	6	6	11

Of the 89 enhancer trap lines, a total of 39 show minimal non-neuronal expression (“neural specific”), and for 50 we mapped and confirmed the transgene integration site (“mapped”). An additional 20 transgenic lines made using defined promoter elements were also imaged as part of this study (Supplemental Figure 3).

a Includes fours lines made using a tph2:Gal4 vector and 1 line made with a dbh:Gal4 construct.

b Each include one line that maps to genomic scaffold in Zv9.

Identification of the genomic location of enhancer trap lines may in some cases reveal valuable information about the cell-type expressing the transgene, or indicate whether an endogenous gene is possibly disrupted by the insertion (Sivasubbu et al., 2006; Bergeron et al., 2015). We therefore collected DNA samples from Gal4 enhancer trap lines, used high-throughput sequencing to map the integration site of the transgenes and verified map positions using PCR (Varshney et al., 2013). For 12 of the 50 imaged lines that were mapped, the integration site was located in an exon or first intron and may therefore disrupt gene expression (Supplemental Table 1). We also confirmed integration positions for an additional 48 lines that were not selected for imaging due to strong non-neuronal expression or the lack of a discrete pattern within the brain. Gene expression is likely perturbed in 21 lines from this group (Supplemental Table 2).

### A 3D searchable interface for finding transgenic lines that express in a target region

All of the transgenic and enhancer trap lines in this dataset have been registered to the same coordinate space, allowing expression patterns to be compared using widely available software tools such as ImageJ or FluoRender (Schneider et al., 2012; Yong et al., 2012). However, searching for lines that show expression in a given 3D region of interest (ROI) or among a specific group of neurons is less straightforward. We addressed this problem by writing new “brain browser” software that allows users to highlight either a given 3D volume, or specific cells within 3D regions, and search for transgenic lines in the data set that contain fluorescent pixels in the defined area of interest.

**Figure 3:**
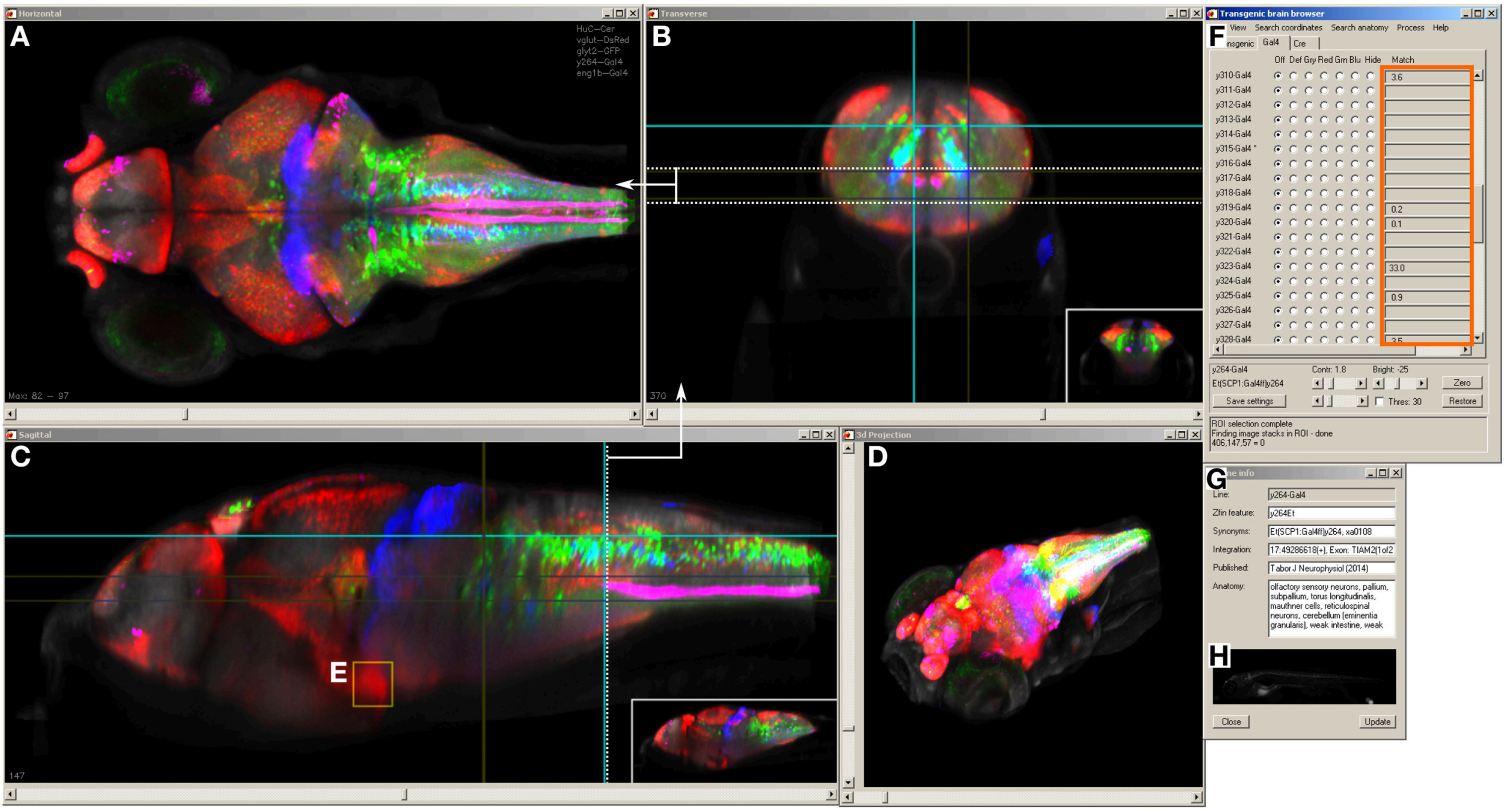
**Brain browser software for searching enhancer trap lines**. The browser displays horizontal **(A)**, transverse (or oblique), **(B)** and sagittal **(C)** views of user-selected image stacks of transgenic and enhancer trap lines. The browser displays single brain slices **(B,C)**, using cross-hairs to indicate the slice location in orthogonal planes (e.g., dotted line in **C** shows the position of the transverse view in **B**). Alternatively, maximum projections of selected regions can be visualized (**A**, between the region indicated by dotted lines in **B**) or a 3D projection of the whole brain **(D)**. The control panel **(F)** lists all lines in the database and during searches, indicates lines (orange box) that label selected 3D regions of interest **(E)** or neuroanatomical structures. Information on the transgene integration site and neuroanatomical annotation are available in an additional panel **(G)**, which also contains epifluorescent images for all Gal4 enhancer trap lines **(H)**.

The Zebrafish Brain Browser simultaneously displays slices or projections of selected lines in horizontal, sagittal, and transverse views (Figures 3A–C). Clicking on a point in any view updates the other two windows to display corresponding slices through the same point. An additional panel gives ancillary information about the currently selected line, including the integration site of the transgene and nearest gene, neuroanatomical annotation as well as an epifluorescent image of the whole fish (Figures 3F–H). Users can rapidly perform maximum projections of selected 3D regions (Figure 3A), zoom into areas of interest, or visualize selected lines as a 3D projection (Figure 3D).

**Figure 4:**
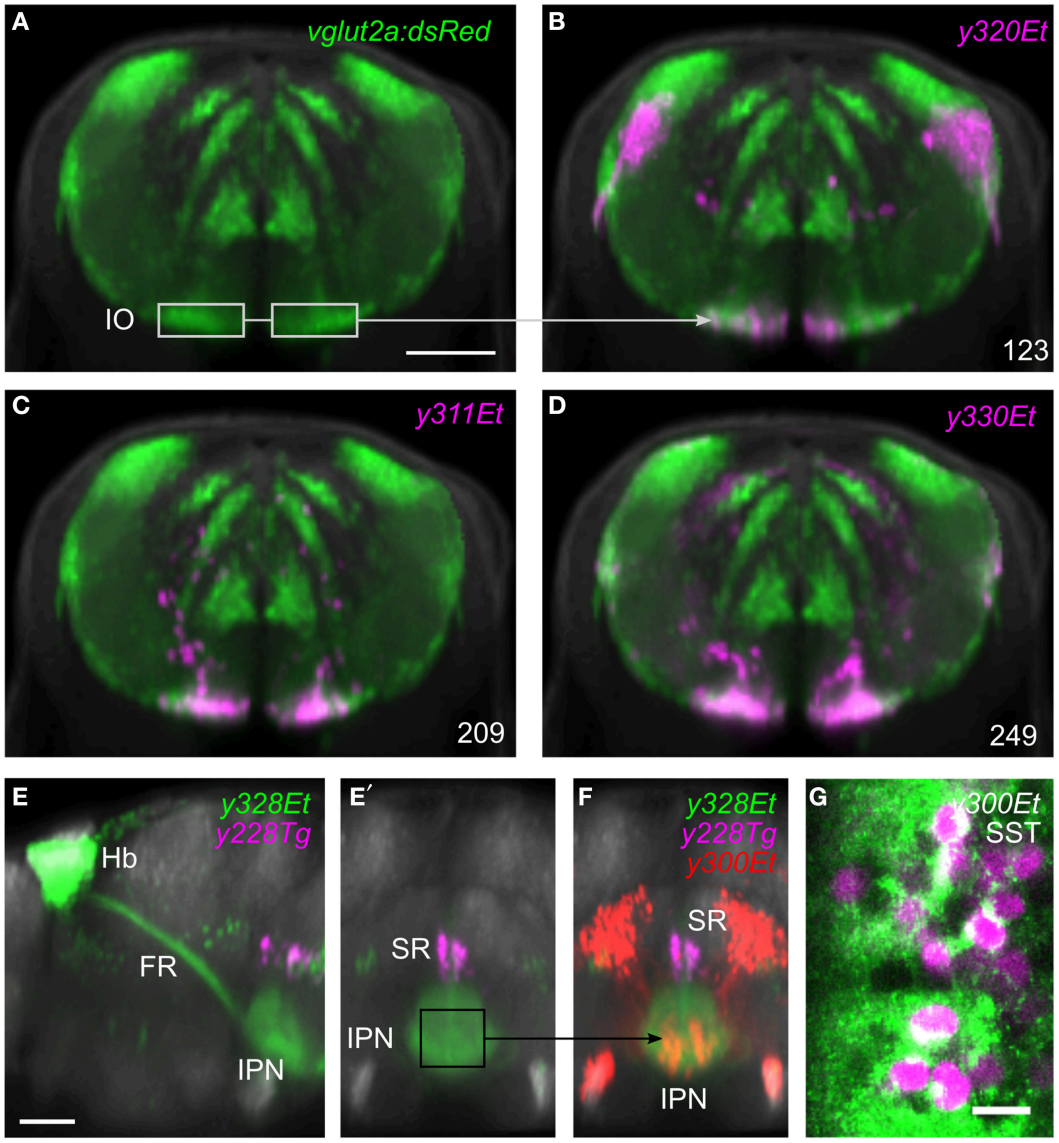
**Region of interest search with the brain browser. (A)** Transverse view through caudal medulla of *vglut2a:DsRed* average brain (green). The boxed regions were used to draw a region of interest around the inferior olive (IO) to search the database for Gal4 lines labeling IO neurons. Scale bar 50 μm. **(B–D)** The three Gal4 enhancer trap lines that most strongly matched the search area (magenta). Each line contained neurons in the region of the IO. **(E)** Sagittal and **(E′)** transverse projections of line *y328Et* (green) which labels the habenula (Hb) and fasciculus retroflexus (FR) including termination zone in the interpeduncular nucleus (IPN). For orientation, a line labeling the superior raphe (SR) is also shown (*y228Tg*, magenta). The termination zone of habenula neurons in the IPN was used to create a 3D search region. Scale bar 50 μm. **(F)** Brain browser image showing the result of the 3D search: enhancer trap line *y300Et* (red) which contained strongly fluorescent cell bodies within the IPN. **(G)** Single confocal plane showing immunostaining against somatostatin (SST, green) and transgene expression in *y300Et* (Kaede fluorescence, magenta) within the IPN. Scale bar 10 μm.

Selecting a single pixel in 3D space highlights all lines that express the transgene at the matching coordinate. To search more thoroughly for a line that labels cells in a given region, the user can create a 3D ROI encompassing a given volume, or select cell bodies that are part of a chosen transgenic line (Figure 3E). The ROI is used as a mask to identify fluorescent pixels in all other lines in the database. Based on the number of fluorescent pixels in the ROI, the user can then inspect lines likely to contain cells in that region. For instance, to identify a Gal4 line that labels the inferior olive (IO), we first located this structure in the ventral caudal medulla using the *vglut2a:DsRed* line (Bae et al., 2009; Satou et al., 2012). We selected an ROI encompassing neurons in the IO (Figure 4A) and inspected Gal4 lines that the browser reported labeled that region (Figures 4B–D). The three top hits, *y311Et, y320Et* and *y330Et* all strongly expressed Gal4 in IO neurons. Similarly, projection targets of axon tracts may be identified by highlighting the termination zone using suitable transgenic lines, and searching for enhancer trap lines containing cells in the region. For instance, line *y328Et* strongly labels the projection of habenula neurons via the fasciculus retroflexus to their termination zone in the interpeduncular nucleus (IPN, Figures 4E,E′). We selected an ROI in the termination area of the fasciculus retroflexus and searched for a Gal4 line that labeled neurons in the IPN. Line *y300Et* contained cell bodies in the search area (Figure 4F) and we confirmed that these neurons are within the IPN by immunostaining *y300Et* for somatostatin (Figure 4G; Hong et al., 2013).

At present, the most widely used neuroanatomical atlas of the larval zebrafish brain contains limited molecularly defined detail for up to 5 dpf larvae (Mueller and Wullimann, 2005) and therefore the neuroanatomical annotation of each enhancer trap line is not complete. Nevertheless, we extracted neuroanatomical terms from the Zebrafish Anatomical Ontology (Sprague et al., 2006) and provide these as search terms from a series of drop-down menus in the browser. Selection of a given term searches neuroanatomical annotations for Gal4 lines, and activates a corresponding ROI that is used to conduct a spatial search of the image data.

### Refining Gal4 reporter domains using cre intersectional expression

We estimated the fraction of the brain labeled by each Gal4 line using a transgenic line with pan-neuronal expression of Cerulean driven by the *elavl3* promoter (*HuC:Cer*) to demarcate the extent of the central nervous system (Supplemental Figure 4). Together, the Gal4 lines included in the database cover 70.5% of the *HuC:Cer* pattern, thus providing experimental access to a substantial fraction of the larval brain. Each line expressed Gal4 in a mean of 1.9% of the brain (median 0.85%), ranging from 0.018% (*y352Et* in which brain expression is primarily driven in bilateral clusters of 10–12 neurons in the caudo-lateral medulla oblongata) to 25.1% (*y365Et* in which expression is driven throughout the neuraxis). These numbers highlight the difficulty in obtaining sufficiently spatially restricted reporter or effector gene expression for functional studies using conventional single transgene approaches. We therefore performed a screen to recover brain-specific Cre enhancer trap lines that could be used with a *UAS:loxP-GFP-loxP-RFP* (*UAS:GR-switch*) transgene to further restrict the expression of a Gal4 activated reporter.

**Figure 5:**
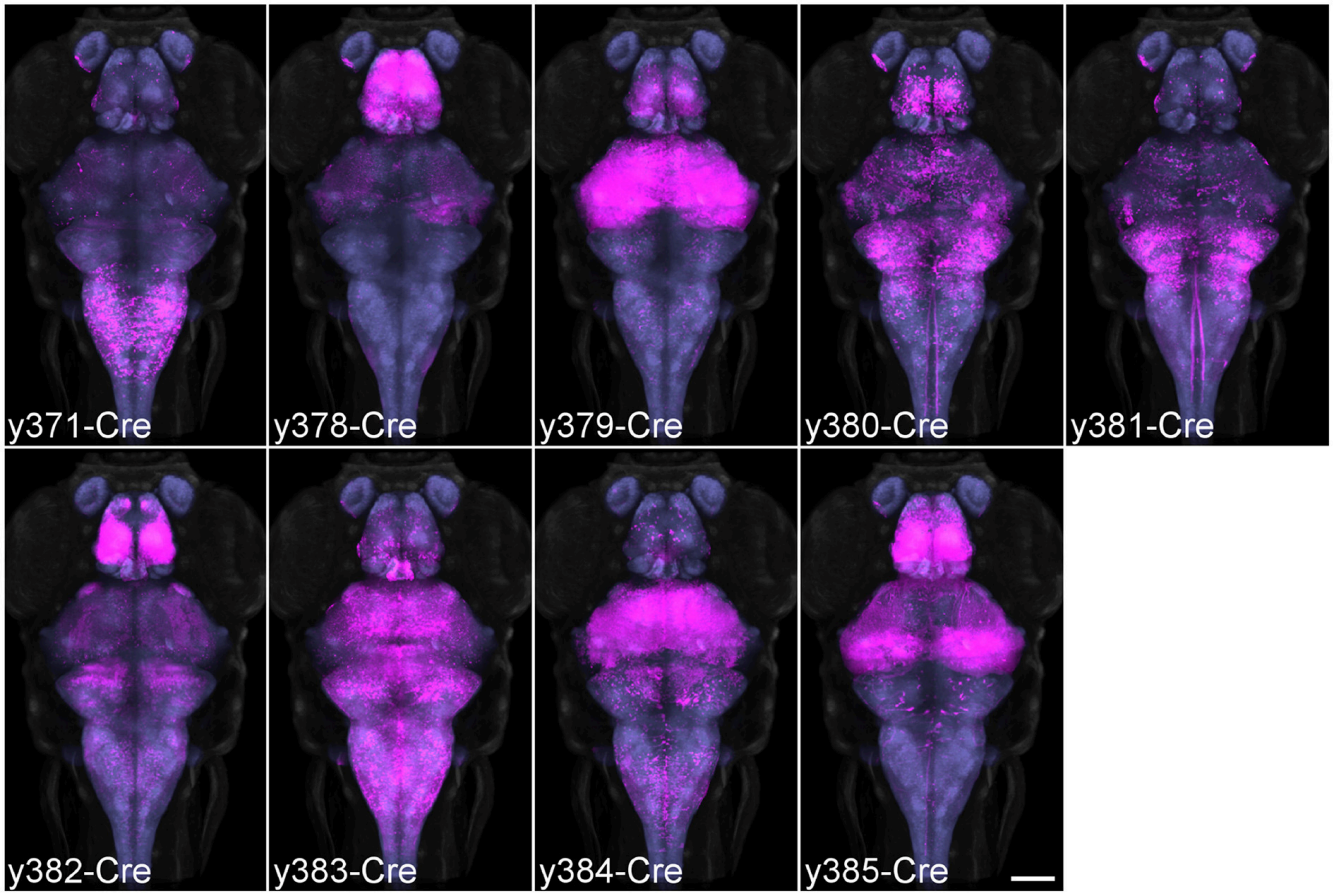
**Average brain representations of Cre enhancer trap lines**. Maximum projections through the 9 Cre enhancer trap lines imaged for this study. Each line was visualized using the β*actin:Switch* transgenic line and scanned with *HuC:Cer* in order to register the pattern to the database. At least three brains were scanned and averaged for each line (magenta). Background: ubiquitous β*actin:Switch* (gray) and pan-neuronal *HuC:Cer* (blue, after brain mask applied). Scale bar 100 μm.

We screened for Cre enhancer trap lines using RFP fluorescence from a *Tg(actb2:loxP-eGFP-loxP-ly-TagRFPT)y272* (β*actin:Switch*) transgenic line (Horstick et al., 2015) and recovered 30 lines after screening 113 founders. We selected nine lines with strong brain expression for high resolution imaging. To register the expression pattern of Cre lines to the same reference space used for our Gal4 lines we used a modification of the pipeline described above. Double transgenic enhancer trap Cre, β*actin:Switch* fish were crossed to the *HuC:Cer* transgenic line. We co-imaged Cerulean and TagRFPT fluorescence, then registered each image stack to the averaged representation of *HuC:Cer* that had been previously transformed onto the *vglut2a:DsRed* reference brain. This enabled us to produce average representations of Cre enhancer trap lines, that could be directly compared with the expression of the Gal4 enhancer trap and transgenic lines previously imaged (Figure 5).

**Figure 6:**
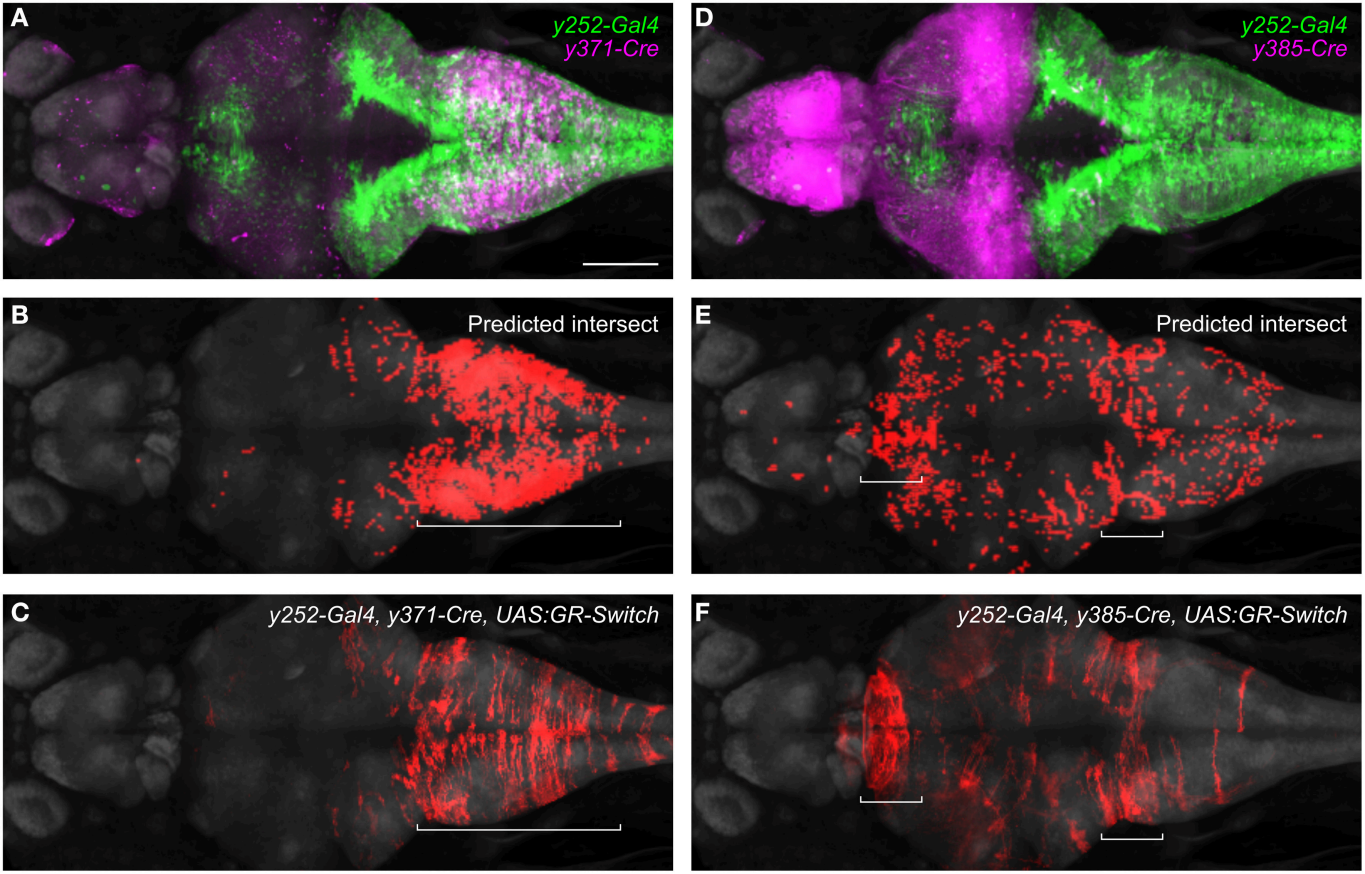
**Intersectional control of reporter gene expression in neurons expressing both Gal4 and Cre. (A)** Expression patterns of *y252-Gal4* and *y371-Cre* superimposed using the brain browser. Scale bar 100 μm. **(B)** Predicted region of co-expression for *y252* and *y371* based on co-localization of strongly fluorescent pixels (red). **(C)** Actual region of co-expression of Gal4 and Cre in *y252-Gal4, y371-Cre, UAS:GR-Switch* embryos. Both predicted and actual expression is primarily confined to the median and caudal medulla. **(D–F)** Similar analysis using *y252-Gal4* and *y385-Cre* in which very strong Cre expression is detected in the forebrain, and weaker Cre expression is observed in the medulla. Both predicted and actual expression is seen in these regions, along with sparse labeling in additional brain regions.

We then used the browser to predict *UAS:GR-switch* reporter expression in crosses between specific Cre and Gal4 lines, by selectively highlighting pixels that contained both Cre and Gal4 expression. Because of our interest in startle modulation, we focused on Gal4 enhancer trap line *y252Et* which labels neurons that regulate startle responses (Bergeron et al., 2015). Two Cre lines (*y371Et* and *y385Et*) differently intersected the expression of Gal4 in *y252Et*, predicting that reporter expression would be constrained to distinct cell populations in triple transgenic larvae. In *y371Et*, Cre expression is confined to part of the medulla (Figure 6A). Imaging of *y252-Gal4, y371-Cre, UAS:GR-switch* larvae revealed that RFP fluorescence was also constrained to the medulla, consistent with the expected co-expression domain of Gal4 and Cre (Figures 6B,C). Conversely, in enhancer trap line *y385Et* Cre is most prominently expressed in the midbrain and forebrain (Figure 6D) and accordingly *y252-Gal4, y385-Cre, UAS:GR-switch* larvae showed RFP fluorescence in the thalamus, with additional scattered RFP+ neurons present in the medulla (Figures 6E,F). Intersectional methods such as these will be essential for highly targeted reporter gene expression and these results further emphasize the value of integrating transgene expression patterns into a unified coordinate system.

### Suppressing non-neuronal expression using microRNA

The majority of the enhancer trap Gal4 and Cre lines in this data set were generated using a vector that strongly enriches for brain-specific expression by inclusion of neuronal-restrictive silencing elements (Bergeron et al., 2012). However, many additional lines are available which contain valuable patterns of expression in the brain accompanied by expression in non-neuronal tissue. Such lines would be of greater utility if the expression outside the brain could be suppressed. We speculated that the addition of specific microRNA target sequences to the 3′ untranslated region of a UAS:reporter transgene would attenuate reporter expression outside the brain, allowing for broader use of these Gal4 lines than is currently possible.

**Figure 7:**
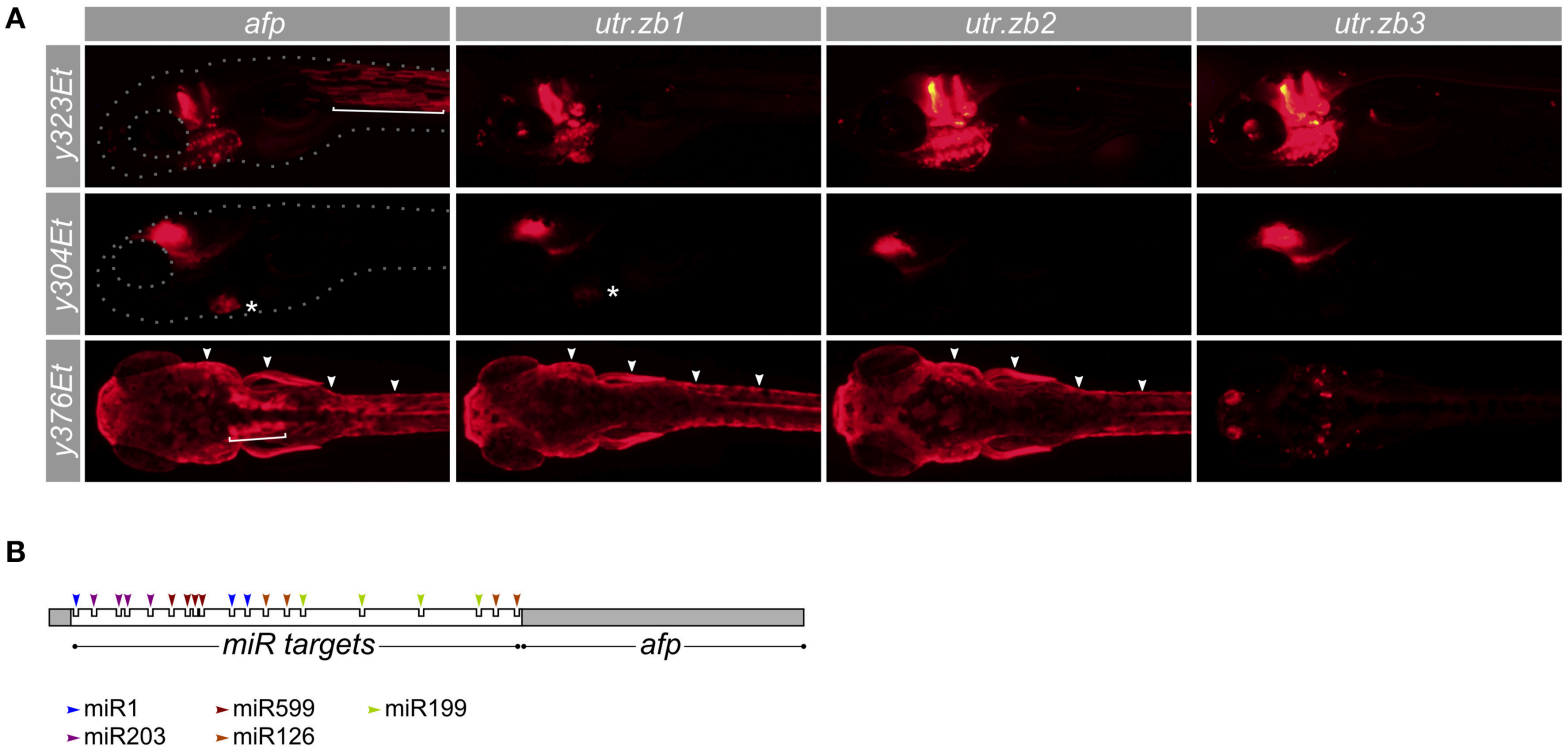
**Suppression of non-neuronal expression using synthetic 3′ untranslated regions. (A)** Development of synthetic utr-zb3 for suppressing Gal4 reporter expression in muscle, skin and heart. We used Gal4 enhancer trap lines *y323Et, y304Et*, and *y376Et* to drive expression in muscle (bracket), heart (asterisk), and skin (arrowheads) respectively—each of these lines also drives expression in the brain. The full pattern of expression is seen using a UAS:epNTR-TagRFPT reporter which has a standard ocean pout anti-freeze protein (afp) 3′ UTR (left panels). Replacing the afp with utr-zb1 eliminates muscle but not heart or skin expression. Similarly utr-zb2 additionally eliminates cardiac expression and utr-zb3 suppresses expression in muscle, heart and skin. **(B)** Schematic of the 3′ untranslated region in utr-zb3. MicroRNA sites are placed at the beginning of the 3′ UTR, ahead of the afp sequence.

MicroRNAs are short hairpin RNA molecules that can greatly reduce the expression of genes with cognate target sequences in the 3′ UTRs of their messenger RNA. While each target site in an mRNA may reduce expression by as much as 50–80%, strongly repressed mRNAs typically contain several target sites in their 3′ UTR (Baek et al., 2008). Many microRNAs are robustly expressed in a tissue specific manner outside the nervous system. We therefore speculated that modifying the 3′ UTR of a UAS:reporter transgene to incorporate multiple target sequences for non-neuronal microRNAs, may lead to reduced expression outside the brain. In zebrafish, microRNA miR-1 is highly expressed in muscle, with little to no expression in the nervous system (Wienholds et al., 2005). Similarly, miR-126 is expressed in heart and miR-199 in epidermis and skeleton, both with minimal neural expression (Wienholds et al., 2005). We constructed a UAS:epNTR-TagRFPT reporter vector, which comprises a fusion of enhanced-potency nitroreductase to TagRFPT followed by the ocean pout antifreeze protein 3′ UTR, which we modified to include miR-1, miR-126, and miR-199 target sites (utr.zb1). Reporter expression was strongly suppressed in both slow and fast muscle but remained in the heart and epidermis (Figure 7A; Supplemental Figure 5). To reduce cardiac expression, we made a new 3′ UTR (utr.zb2) incorporating additional target sites for miR-499 which is highly expressed in the heart (Nachtigall et al., 2014). Transgenic larvae with the utr.zb2 showed suppression of reporter expression in the heart in addition to the suppression in muscle observed with utr.zb1. As these larvae retained reporter expression in skin, we added target sequences to the 3′ UTR for miR-203a which is expressed in epidermis (Wienholds et al., 2005). Transgenic *Tg(UAS:epNTR-TagRFPT-utr.zb3)y362* larvae with this 3′ UTR (utr.zb3; Supplemental Figure 6) showed a strong reduction in expression in muscle, heart, and skin with no apparent reduction in brain expression (Figures 7A,B). Accordingly, we anticipate that *utr.zb3* will expand the usefulness of a wide range of existing Gal4 lines.

## Discussion

Binary transgenic expression systems such as Gal4/UAS are powerful tools for dissecting neuronal circuitry and consequently, enhancer trap screens have provided the zebrafish community with many valuable Gal4 lines. Here we offer a solution to a practical obstacle to working with Gal4 lines: the difficulty in finding a line that labels a specific population of neurons. The 80 Gal4 lines presented in this database were registered onto a common reference brain, and, using our browser software, can be easily searched to identify a line that labels a specific brain region.

The pipeline we have established for registering brains into a common reference space produces average brain images that are accurate to around one cell diameter, which is similar to the accuracy achieved by two other large-scale confocal registration projects in larval zebrafish (Ronneberger et al., 2012; Randlett et al., 2015). Because our confocal images were acquired from a single orientation, there is pronounced fluorescence attenuation in the Z-dimension due to effects of absorption and light-scattering. For example, the resolution in our image stacks is notably less clear in the hypothalamus, than in dorsal regions such as the tectum. This problem was addressed in an earlier study in zebrafish that developed ViBE-Z software for image registration (Ronneberger et al., 2012). ViBE-Z achieves highly accurate reconstruction of brain expression by combining a light attenuation model with dual high and low intensity brain scans at four positions, and thus requires significant imaging time per embryo. In addition, ViBE-Z was limited to 2–4 dpf brains, whereas most larval zebrafish behavioral studies are performed at 5–7 dpf. Thus, as for the Z-Brain database (Randlett et al., 2015), we imaged larvae at 6 dpf. Whereas, Z-Brain used antibody staining in fixed tissue, our pipeline used live imaging of fluorescent transgenic reporters. Although we recognize that live imaging limits the scope of available markers, it avoids artifacts associated with fixation and staining and increases throughput. Indeed, by employing an optimized scanning protocol that required only two image stacks per embryo, image acquisition time for each transgenic line (for the minimum of three larvae) was 120 min, and using a parallel computing resource, an additional 60 min for image processing. An additional strength of Z-Brain is an extensive effort at manual segmentation of neuroanatomical regions, which facilitates annotation. Our preliminary studies suggest that by imaging larvae co-stained for multiple reference channels (e.g., DsRed and total ERK in vglut2a:DsRed larvae) it is feasible to align expression data acquired from live imaging and immunostaining. Thus, data from independent registration projects performed at the same developmental stage may be merged, and take advantage of the complementary strengths of different approaches.

The brain browser software provides a simple way to identify enhancer trap Gal4 lines that label a specific 3-dimensional region of the brain. To assist with this, the current data set includes 20 transgenic lines that were generated with defined promoters or BAC transgenesis that reproduce known neuroanatomical expression patterns of specific genes. These lines provide landmarks for interpreting expression patterns in the enhancer trap lines, as illustrated above by the use of ventral medulla *vglut2a:DsRed* expression to identify Gal4 lines that label the inferior olive. The database we present is easily extendable: new lines can be added simply by copying registered image stacks to the browser data directory and manually annotated within the browser. The browser can search the neuroanatomical annotations, using terms from the zebrafish anatomical ontology. For several search terms (e.g., inferior olive) we have generated a mask that is additionally used to conduct a spatial search for lines with fluorescence in the corresponding region. Such masks are ROIs that can be created from within the browser and can be easily generated by users of the software, providing the possibility of expanding and refining spatial annotation as more detailed neuroanatomical information becomes available (Randlett et al., 2015).

A major impetus for this work has been our effort to conduct circuit breaking screens in larval zebrafish. Circuit breaking, or “neurotrapping” screens which use libraries of Gal4 lines to inactivate cohorts of neurons are a standard method in *Drosophila* for identifying behaviorally relevant circuitry (Pitman et al., 2006; Pool et al., 2014). Such screens are also feasible in zebrafish (Bergeron et al., 2015), however they require maintaining a large number of transgenic lines. In principle, a more systematic screen for behaviorally relevant neurons could be performed, by using a small set of transgenic lines with non-overlapping expression patterns that together would target every neuron in the brain. A hit from this initial set could then be interrogated using either a second set of Gal4 lines that subdivide the initial expression domain or by using Cre lines that further constrain reporter expression. To facilitate testing of a “nested” set of lines, the brain browser can automatically delineate a set of lines that together, subdivide the expression domain of any given selected line. For instance, using the *HuC:Cer* transgenic line image as a template, the browser identifies a set of eight lines that together express Gal4 in 50% of the brain with minimal overlap. This set of lines may be a valuable starting point for screening lines with a functional role in behavior. An important caveat is that while neurons labeled in most enhancer trap lines show reproducible overall morphologies, the actual locations of specific cells may differ between individuals. Thus, in some cases enhancer trap lines that appear to overlap in a discrete brain region may nevertheless label closely intermingled but distinct neuronal cell types. Thus, while computational predictions of overlapping expression may be useful for selecting transgenic lines, they will require empirical validation.

Transgenic technologies alone do not yet enable neuroscientists to interrogate the nervous system on a cell-by-cell basis. Most transgenic lines have expression in thousands of cells, and more often than not, this expression is not limited to the nervous system. This study provides partial solutions to these two problems. We confirm that Cre enhancer trap lines can successfully constrain Gal4 expression domains, and have designed a novel UAS transgenic line that suppresses reporter expression in heart, muscle and skin. Using the *UAS:GR-switch* reporter, the Cre enhancer trap lines presented here enable Gal4 expression domains to be limited to cells co-expressing Cre and Gal4. With a larger set of Cre lines, obtained by further screening or incorporation of existing resources (Jungke et al., 2015), Gal4 expression domains may be more systematically subdivided.

Robust expression outside the brain limits the experimental utility of many enhancer trap lines. Previously, we attempted to address this by constraining transgene expression to the nervous system through the incorporation of neuronal restrictive silencing elements (NRSEs; Bergeron et al., 2012). Indeed, by incorporating two NRSE motifs (the REx2 element) into our Gal4 enhancer trap vector, we achieved a five-fold increase in the recovery of enhancer trap lines with brain-specific Gal4 expression. While this approach allows for the generation of new brain-specific enhancer trap lines, there are many existing Gal4 lines with extremely useful expression patterns in the brain that are accompanied by strong expression in non-neuronal tissue. To better utilize existing Gal4 lines that lack NRSEs, we attempted to make a brain-specific Gal4 reporter by incorporating the REx2 element into the promoter or intron of a UAS:mCherry transgene. Unfortunately, this was ineffective at suppressing non-neural mCherry expression. Here we describe a different strategy to reduce UAS reporter expression outside the brain, by constructing a synthetic 3′ untranslated region that contains target sites for microRNAs that are strongly expressed in muscle, heart and epidermis. Several microRNAs have been reported that are strongly expressed in these tissues with low or undetectable expression in the larval brain. By incorporating 3–4 target sequences for each such microRNA into the 3′ untranslated region of a UAS reporter transgene, expression was strongly suppressed in heart, muscle, and epidermis. Importantly, expression in the brain was not also reduced. In contrast, we noticed an apparent increase in expression for the utr.zb2 and utr.zb3 containing transgenes, which we attribute to differences in the site of transgene insertion. This new method expands the usefulness of a wide range of existing Gal4 lines, enabling such lines to be used for ablation, bioluminescent imaging, or optogenetic experiments. However, expression is only suppressed in the specific cell-types that express high levels of the microRNAs that we selected at larval stages. Thus, a limitation of our current best synthetic 3′ UTR (utr.zb3) is that it does not suppress expression in the notochord and suppression in the heart is most robust after 5 dpf. We will continue to test microRNA target sequences that may more strongly and broadly suppress reporter expression outside the brain.

Current genetic methods already enable both non-invasive visualization and functional interrogation of neurons. However, further refinements of transgenic technologies will allow for more precise selective targeting of neuronal cell populations. The new Gal4 and Cre transgenic reporter lines described here, together with our browser software will therefore facilitate the use of zebrafish in mapping the neuronal circuits that control physiology and behavior.

## Materials and methods

### Software and data availability

The transgenic brain browser is written as IDL runtime code and can run under the IDL Virtual Machine which is freely available (www.exelisvis.com). The software, reference brain, registered brain averages and epifluorescent images can be downloaded from our website (https://science.nichd.nih.gov/confluence/display/burgess/Software). Download and extract the zbb.zip file. Instructions for the operation of the browser are included in the download. Epifluorescent images and mapping data for enhancer trap lines are available at zfin.org.

### Animal husbandry

The Gal4 and Cre enhancer trap lines in this study were maintained on a Tubingen long fin strain background. Embryos were raised in E3 medium supplemented with 1.5 mM HEPES pH 7.3 (E3h) at 28C on a 14 h:10 h light:dark cycle with medium changes at least every 2 days. All *in vivo* experimental procedures were conducted according to National Institutes of Health guidelines for animal research and were approved by the NICHD animal care and use committee.

### Transgenic lines

Seventy-four of the Gal4 enhancer trap lines described here were generated by our laboratory as previously described (Bergeron et al., 2012). Briefly, either cfos or SCP1 basal promoter sequences were used together with the Gal4 variant Gal4ff. Most lines also included a sequence derived from juxtaposing two neuronal restrictive silencing elements (the REx2 motif) in order to restrict expression to the nervous system. Six additional Gal4 lines were recovered by chance: we observed a strong position effect while generating transgenic lines using the tryptophan hydroxylase and dopamine beta hydroxylase promoters (Yokogawa et al., 2012), necessitating the screening of multiple founders to isolate a line with the expression pattern matching that of the endogenous gene. Lines with robust expression in the brain outside the expected domain are thus included as enhancer traps in this paper. Since most founder fish contained several tol2-mediated integrations that resulted in multiple overlapping patterns of expression, fish were outcrossed over at least four generations in order to isolate transgenic lines with single reproducible expression patterns that segregated in Mendelian ratios. Enhancer trap Gal4 expression patterns were maintained and visualized using the *Tg(UAS-E1b:Kaede)s1999t* (UAS:Kaede) reporter line (Davison et al., 2007). Other transgenic Gal4 fish were visualized using *Tg2(14xUAS:GFP)nns19* (Kimura et al., 2013).

For the Cre enhancer trap screen, we used the REx2-SCP1 synthetic promoter that we previously created for Gal4 enhancer trapping (Bergeron et al., 2012), as a basal promoter for a fusion gene containing zebrafish-optimized (zf1) Cre and Cerulean linked by a porcine teschovirus-1 2A peptide to yield separate proteins (Cre.zf1-2a-Cer.zf1; Provost et al., 2007; Horstick et al., 2015). The REx2-SCP1:Cre.zf1-2a-Cer.zf1 cassette was placed in a mini-tol2 backbone (Urasaki et al., 2006) and injected with tol2 to generate founders. Some additional Cre lines were based on a modification of this vector, where we added 60 bp *attP* sites flanking the transgene cassette (inside the tol2 arms; Groth et al., 2000). In principle the presence of *attP* sites will enable the Cre trap to be replaced by another reporter using PhiC31 recombinase-mediated cassette exchange (Hu et al., 2011), similar to the InSite system used in *Drosophila* (Gohl et al., 2011), however we have not yet tested the efficiency of reporter replacement. We initially screened for founders using the Cerulean protein in the vector; however, this was not successful, presumably because the Cerulean fluorescence was in most cases too dim to detect with an epifluorescent microscope. Instead, we screened and maintain lines using the β*actin:Switch* transgenic line (*Tg(actb2:loxP-eGFP-loxP-ly-TagRFPT)y272*) previously described (Horstick et al., 2015).

The plasmid used to make *Tg(elavl3:ubci-Cer-sv40)y342* (*HuC:Cer*) uses a 3.1 kb fragment from the elavl3 promoter (Park et al., 2000; Kimura et al., 2008) to drive pan-neuronal expression of Cerulean fluorescent protein and includes the ubiquitin C intron for stronger expression (Horstick et al., 2015). As the optimized woodchuck hepatitis virus post-transcriptional element (oPre) does not increase expression levels in zebrafish (Horstick et al., 2015), we used inverse PCR to remove oPre from the UAS:BGi-epNTR-TagRFPT-oPre plasmid which contains enhanced-potency nitroreductase (epNTR) fused to TagRFPT and the rabbit β-globin intron (BGi) for stronger expression (Tabor et al., 2014). We then generated a new transgenic line for ablation studies *Tg(UAS-E1b:BGi-epNTR-TagRFPT)y361*. To target noradrenergic neurons, we isolated a 3007 bp fragment from the dopamine beta-hydroxylase gene, including promoter and first exon up to the ATG (chr10:10572957-10575963) from BAC.CH211-270H11, which we subcloned into pT2MCSkG4FF (Bergeron et al., 2012), then used tol2 transgenesis to generate founders. *Tg(-3.0dbh:Gal4ff)y360* shows expression in the dorsal caudal hindbrain consistent with the position of noradrenergic neurons of the vagal area (Tay et al., 2011). To make the UAS:BGi-lox-emGFP.zf1-lox-lyn-TagRFPT-afp plasmid, we PCR amplified codon optimized emerald GFP (emGFP; Bergeron et al., 2015), adding loxP sites and recombined the product into UAS:BGi-lynTagRFPT (Yokogawa et al., 2012) using SLiCE (Zhang et al., 2012). This plasmid was injected with tol1 transposase to make the line *Tg(UAS:BGi-loxP-eGFP.zf1-loxP-lyn-TagRFPT)y363* (*UAS:GR-Switch*). *Tg(evx2:Gal4)nns52* and *Tg(shox2:Gal4)nns51* lines were generated by CRISPR/Cas9-mediated knock-in (Kimura et al., 2014) using donor constructs that either contained (evx2:Gal4) or did not contain the hsp70 promoter (shox2:Gal4). A target sequence for sgRNA of GGGGCTCGCGGTGAGGGAAGG (−78~-90) was used for evx2:Gal4, while GGCGGCAGCTGAGCTGAATGCGG (+274~+276) was used for shox2:Gal4.

Other transgenic lines used were: *Tg(tph2:Gal4ff)y228* (Yokogawa et al., 2012), *Tg(-3.2fev:EGFP)ne0214* (pet1:GFP; Lillesaar et al., 2009), *TgBAC(slc17a6b:loxP-DsRed- loxP-GFP)nns14* (vglut2a:DsRed; Satou et al., 2012), *Tg(slc6a3:EGFP)ot80* (dat:GFP; Xi et al., 2011), *Tg(kdr:GFP)la116* (flk:GFP; Choi et al., 2007), *Tg(atoh7:GFP)rw021* (ath5:GFP; Masai et al., 2003), *Tg(gfap:GFP)mi2001* (gfap:GFP; Bernardos and Raymond, 2006), *Tg(slc6a5:GFP)cf3* (glyt2:GFP; McLean et al., 2007), *Tg(-17.6isl2b:GFP)zc7* (isl2b:GFP; Pittman et al., 2008), *Tg(phox2b:EGFP)w37* (phox2b:GFP; Nechiporuk et al., 2007), *TgBAC(vsx2:Gal4ff)nns18* (chx10:Gal4; Kimura et al., 2013), *Tg(eng1b:Gal4)nns40* (eng1b:GFP; Kimura et al., 2014), *TgBAC(gsx1:GFP)nns32* (gsx1:GFP), *TgBAC(gad1b:GFP)nns25* (gad1b:GFP; Satou et al., 2013) and *Gt(T2KSAG)j1229* (Burgess et al., 2009).

### Imaging

Lines included in our database were crossed to *vglut2a:DsRed*, which broadly labels glutamatergic neurons throughout the brain (Kinkhabwala et al., 2011; Satou et al., 2012), providing a suitable channel for image registration. Embryos from these crosses were raised in E3h media containing 300 μM N-Phenylthiourea (PTU) starting at 8–22 h post fertilization (hpf) to suppress melanophore formation and sorted for fluorescence at 2 days post fertilization (2 dpf). An inverted laser-scanning confocal microscope (Leica TCS SP5 II) equipped with an automated stage and 25x/0.95 numerical aperture apochromatic water immersion lens (Leica # 11506340) was used to acquire confocal stacks of transgenic fish and immunofluorescently labeled samples. Live larvae were anesthetized in 0.24 mg/mL tricaine methanesulfonate (MS-222) for 3 min prior to mounting at 6 dpf. Live or fixed embryos were then mounted in 2.5% low melting point agarose placed in a 3D printed ABS four-well plastic insert (Stratasys uPrint) within a cell culture chamber with a number 1.5 thickness (0.17 ± 0.005 mm) cover glass bottom (Lab-Tek II 155379). A 488 nm argon laser line and a 561 nm diode-pumped solid state (DPSS) laser were used to excite fluorophores with images acquired as serial sections along the z-axis at 2.0 μm intervals between dorsal and ventral surfaces of the brain in a 1 × 2 tiled array to visualize the brain and a portion of the spinal cord. To shorten scan duration and allow for laser compensation over the z-dimension, channels were acquired simultaneously at a 1 × 1 × 2 μm; xyz pixel spacing. Laser illumination was increased via acousto-optic tunable filter with z-position to counter attenuation with ramp points set every ~70 μm. Fluorescent emission from the 488 nm excitation was collected with a hybrid detector with a spectral window of 500–550 nm and emission from the 561 nm excitation collected by a second hybrid detector set at 571–700 nm. In order to minimize the cross-channel contamination present between imaged fluorophores, dye separation was performed in the Leica acquisition software (Leica Application Suite—Advanced Fluorescence, LAS AF) with coefficients based on published spectra of imaged fluorophores and the spectral windows used for acquisition. Resulting rostral and caudal stacks were stitched together (Preibisch et al., 2009) and channels split in Fiji (Schindelin et al., 2012) prior to registration.

### Registration

The Computational Morphometry Toolkit (CMTK) *convertx, registrationx, warpx, reformatx, average_images*, and *levelset* commands were used to process and register image stacks (http://www.nitrc.org/projects/cmtk/). As reference choice can impact the quality of registration, we used the normalized cross-correlation (NCC) between duplicate scans of the brains from a “calibration set” (i.e., two *tph2:Gal4* and two *y307Et* larvae) to identify the best performing reference as well as optimal registration parameters. We reasoned that successive scans of the same fish, initially and following removal and remounting, would approximate the ideal registration case (i.e., alignment of an identical underlying pattern), and that use of the best reference and optimal registration parameters should maximize the NCC. As potential optimal reference brains, we tested the *vglut2a:DsRed* channels from the scans of 255 enhancer trap and transgenic fish as well as iteratively shaped averages derived from the top 5, 10, and 20 performing channels.

We first registered all eight scans (i.e., the two scans for each of the four fish in the calibration set) to each potential reference brain. Then, for each reference brain, we computed the NCC for both the vglut2a:DsRed channel and the Gal4/Kaede channel for all four calibration larvae (i.e., the first DsRed scan vs. the second DsRed scan for fish #1, the first Kaede scan vs. the second Kaede scan for fish #1, etc for a total of eight comparisons per reference brain). We then ranked reference brains by the mean of the four NCCs for the vglut2a:DsRed comparison (Supplemental Figure 1A) and the mean NCC for the Gal4/Kaede comparison (Supplemental Figure 1A′). As expected, the vglut2a:DsRed average NCC and the Gal4/Kaede average NCC were correlated (not shown). In computing the NCC for the vglut2a:DsRed channel, we were able to calculate a more stringent NCC by excluding pixels outside the expression pattern, by using the reference brain vGlut2a:DsRed pattern as a mask. This procedure, however, could not be applied to the Gal4/Kaede channel because the Gal4/Kaede expression does not overlap entirely with the vGlut2a:DsRed pattern. Therefore, the NCC values in Supplemental Figure 1A′ are not masked. Averaged brains performed worse than their constituents (Supplemental Figure 1A). The specific reference brain chosen for all subsequent registrations had the highest rank among the 255 vglut2a:DsRed brains, was 9th ranked for Kaede comparisons, and was close to symmetrically oriented in horizontal and transverse sections.

Next, we used the calibration set to systematically test and identify parameters that yielded the most accurate registrations (e.g., Supplemental Figure 1B) while minimizing computation time when the impact on accuracy was negligible (e.g., Supplemental Figures 1C,D). Parameters were adjusted in isolation and the NCCs and walltimes recorded. A full list of the settings we tested and the parameters we recommend for registration of similar confocal stacks with *vglut2a:DsRed* as a reference are listed in Supplemental Table 3.

For registrations using *vglut2a:DsRed* as a reference, “—dofs 12—min-stepsize 1” was used for the initial rigid alignment while “—fast—grid-spacing 100—smoothness-constraint-weight 1e-1—grid-refine 2—min-stepsize 0.25—adaptive-fix-thresh 0.25” was used for the subsequent nonrigid registration. For *Cre* enhancer trap lines, *HuC:Cer* was used as the reference pattern for registration instead. For these registrations, the same rigid parameters were used (i.e., “—dofs 12—–min-stepsize 1”) while “—fast—grid-spacing 100—smoothness-constraint-weight 1e-1—grid-refine 0—min-stepsize 0.25—adaptive-fix-thresh 0.25” was used for the *HuC:Cer* nonrigid registration. For the intersection of Cre lines to *y252*, the *y252* mean was used as a reference and a rigid registration was performed without an additional nonrigid component. Resulting image stacks were normalized and averaged using the *average_images* command. To mask images, an initial mask was generated with CMTK's *levelset* command from binarizations of the mean of the *HuC:Cer* and *vglut2a:DsRed* scans. This initial mask was then manually expanded to encompass regions with weak *HuC:Cer* transgene expression such as the retina and pituitary and refined in order to minimize the inclusion of skin and other non-neural tissue in problematic lines. For example, lines with weak fluorescence required higher laser levels which often resulted in greater reflectance from skin. Finally, we linearly adjusted pixel intensities to saturate the top 0.01% of pixels in each averaged image stack for visualization and analysis.

### Annotation

Neuroanatomical annotation was based primarily on Mueller and Wullimann (2005), using the *HuC:Cer* and *vglut2a:DsRed* transgenic line channels in the brain browser as anatomical references. Due to the nature of transgene expression patterns, in some cases, patterns may be broader than the corresponding neuroanatomical region annotated. We used terms from the Zebrafish Anatomical Ontology where possible, supplemented by more up to data terminology where available (Amo et al., 2010; Mueller, 2012).

### Synthetic 3′ UTR constructs

To make utr.zb1, we designed a DNA fragment with target sequences for miR1-3p, miR126-3p, and miR199-5p. MicroRNA targeting is primarily driven by the 7 bp seed sequence, however context outside this region may influence pairing (Bartel, 2009). For each microRNA, we therefore used TargetScanFish (release 6.2; Ulitsky et al., 2012) to identify an endogenous zebrafish transcript with a 3′ UTR enriched in its predicted target sequences: respectively *tagln2* (miR1), *clcn6* (miR126), and *BX927290.1* (miR499). Regions of each gene's UTR that contained multiple microRNA targets were combined into a single 472 nucleotide sequence, which we then edited to remove all other microRNA targets and to make synthesizable. We synthesized this sequence as a gblock (IDT) and cloned it into the HpaI site in the ocean pout antifreeze protein 3′ UTR contained in UAS:BGi-epNTR-TagRFPT. This was used to make *Tg(UAS:BGi-epNTR-TagRFPT-utr.zb1)* using tol1 transgenesis using tol1.zf1 mRNA (Koga et al., 2008; Horstick et al., 2015). For utr.zb2 we searched TargetScanFish for a 3′ UTR enriched in target sequences for miR-499-5p, which is strongly expressed in zebrafish heart (Nachtigall et al., 2014). We synthesized a 68 nucleotide fragment from the *shroom1* gene, edited it to remove other microRNA targets and cloned it into UAS:BGi-epNTR-TagRFPT-utr.zb1. This was used to make *Tg(UAS:BGi-epNTR-TagRFPT-utr.zb2).* Next we added target sequences for miR-203a-3p, which is strongly expressed in zebrafish epidermis (Wienholds et al., 2005). A 105 nucleotide sequence with multiple miR203 targets was copied from the *dntt* gene 3′ UTR, and cloned into UAS:BGi-epNTR-TagRFPT-utr.zb2 (see Supplemental Figure 6 for the annotated sequence of utr.zb3). The resulting plasmid was used to make *Tg(UAS:BGi-epNTR-TagRFPT-utr.zb3)y362*.

### Transgene insertion site mapping

Insertion site mapping for Gal4 enhancer trap lines was performed as described previously (Varshney et al., 2013) with the following modifications. Genomic DNA was digested with Mse1 and Bfa1 and barcoded linkers ligated to digested products. The first round of PCR was performed using Tol2 ITR primer 5′-AAT TTTCCCTAAGTACTTGTACTTTCACTTGAGTAA and a linker primer 5′-GTA ATACGACTCACTATAGGGCACGCGTG using cycle conditions: 95°C 120 s, 25 cycles of: 95°C 15 s, 55°C 30 s, 72°C 60 s. PCR amplicons from the first round were diluted 1:50 and a second round of PCR was performed using a nested Tol2 ITR primer : 5′-TCACTTGAGTAAAATTTTTGAGTACTTTTTACACCTC and nested linker primer 5′-GCGTGGTCGACTGCGCAT with cycle conditions: 95°C 120 s, then 20 cycles of: 95°C 15 s, 58°C 30 s, 72°C 60 s. Amplicons from the second round were pooled and the sequencing library was prepared for the Illumina Miseq sequencing platform. The insertion sites were mapped using GeIST mapping pipeline (LaFave et al., 2015). We then used Primer3 (Rozen and Skaletsky, 2000) to design primers against the flanking region (outside the sequence obtained by high throughput sequencing) and performed PCR to verify the insertion sites.

## Author contributions

GM, KT, and HB conceived and designed the project. GM, KT, and MB performed image acquisition and registration. KT, JS and SH generated transgenic lines. JS, GK, MF, and SB identified integration sites. TM and HB annotated lines. GM and HB wrote software. GM and HB wrote the manuscript.

### Conflict of interest statement

The authors declare that the research was conducted in the absence of any commercial or financial relationships that could be construed as a potential conflict of interest.

## Supplementary material

Supplemental Table 1 **Neuroanatomical annotation and integration site mapping for Gal4 enhancer trap lines imaged as part of this study**. Annotations use terms from the zebrafish anatomical ontology, except where available terms are insufficiently specific. Lines which subjectively appear to most prominently label a single neuroanatomical structure are annotated as ‘selective’. Many lines include expression in retina and/or spinal cord. Because of the limitations of our confocal and epifluorescent analysis, we have not generally included further annotation of cells within these structures. We also provide limited annotation of structures outside the nervous system. Abbreviations: anterior lateral line ganglion, aLLg; nucleus of the medial longitudinal fasciculus, Nuc MLF; posterior lateral line ganglion, pLLg. Genes with accession numbers “ENS…” can be queried at Ensembl (http://www.ensembl.org). ^*^ Line *y372Et* contains a second linked integration in *atp11a* at Chr1:46822150.

Supplemental Table 2 **Anatomical annotation for additional Gal4 lines with verified integration sites**. These lines were not used for high resolution imaging in this study. For each line, at least two lines of evidence support the integration site: either high throughput mapping and targeted PCR confirmation, or high throughput mapping and independent mapping data obtained using linker mediated PCR.

Supplemental Table 3 **Summary of recommended CMTK parameters for registration of image stacks acquired using live *vglut2a:DsRed* fluorescence as the reference channel**.

Supplemental Figure 1 **Optimization of brain registration using CMTK. (A)** Normalized cross-correlation (NCC) for duplicate confocal scans of the vglut2a:DsRed channel in each of four brains in the calibration set (blue: *tph:gal4* fish 1; red: *tph2:gal4* fish 2; green: *y307Et* fish 1; purple: *y307Et* fish 2), after registration by full affine transformation to each of 255 vglut2a:DsRed expressing reference brains. Empty pixels were excluded from the computation using the reference brains as a mask. For each reference, the mean of the four NCCs was taken, and used to provide a rank (higher ranks to the right). In addition, the top 5, 10, and 20 reference brains (based on mean NCC) were used to generate iterative shaped averages (a5, a10, and a20 respectively) whose performance as a reference was also assessed. **(A′)** NCC for the Gal4/Kaede channel from the same set of calibration and reference brains, however no mask was used before the NCC computation (see Methods). Arrows in **(A,A′)** indicate the reference brain we selected to use for subsequent registrations. **(B)** Comparison of the average NCC of the calibration set using 9° of freedom rigid transformation (9: translation, rotation, and anisotropic scale), full affine transformation (12: translation, rotation, anisotropic scale, and sheer), and affine plus warp transformation (W: translation, rotation, anisotropic scale, sheer, and nonrigid). Line colors indicate larvae as in **(A)**.While a number of registration parameters were optimized to maximize the average NCC of the calibration set (see Supplemental Table 3 for full list of tested and recommended parameters), a number of parameters showed no improvement in the average NCC of the calibration set, and so settings that minimized CPU walltime were selected instead. **(C)** Rigid registrations showed negligible improvement in NCC beyond a *min-stepsize* of 1 while greatly increasing the CPU time. Thus, a *min-stepsize* setting of 1 was used for registrations. **(D)** Nonrigid registrations showed negligible improvement in NCC beyond a *min-stepsize* of 0.25 while greatly increasing the CPU time. Thus, a *min-stepsize* setting of 0.25 was used for registrations.

Supplemental Figure 2 **Assessing registration accuracy by co-localized Gal4 reporter gene expression**. Dorsal **(A)** and transverse **(B)** views of brain browser projections for enhancer trap line *y264Tg* (green) and transgenic line *j1229aGt* (magenta) in the region including the Mauthner cells (arrowheads). **(C,C′)** Dorsal view of maximum projection of average representations of *y339Et*, made from two independent sets of three embryos each. Dotted line shows location of transverse slice shown in **(D)**. **(D)** Slice through the midbrain and diencephalon of superimposed *y339Et* averages. In the right optic tectum, a neuropil layer (arrows) in the averages is misaligned by 1–2 cell diameters. **(E)** Horizontal view of a slice through the hindbrain from *HuC:Cer* average brain. Arrowheads indicate gaps in the tissue lacking neuronal Cer expression. *flk:GFP* expression, which labels blood vessels, is superimposed in **(F)**. Anterior (A), Posterior (P), Dorsal (D), Ventral (V). All scale bars 50 μm.

Supplemental Figure 3 **Transgenic lines providing neuroanatomical markers**. Maximum projections **(A)** and expression domains **(B)** of transgenic lines that were imaged as part of this study. Gal4 lines were visualized using *UAS:GFP* (magenta). Background: ubiquitous β*actin:Switch* (gray) and pan-neuronal *HuC:Cer* (blue, after brain mask applied).

Supplemental Figure 4 **Extent of brain expression in Gal4 enhancer trap lines. (A)** Histogram of the extent of brain coverage for the 80 Gal4 enhancer trap lines imaged in this study. **(B)** Sagittal hemi-projection for the line with the most restricted Gal4 expression pattern *y352Et*, for two lines with close to the median extent of Gal4 expression (*y236Et* and *y353Et*) and for the line with the broadest Gal4 expression pattern included in the database, *y365Et*.

Supplemental Figure 5 **Synthetic 3′ UTR *utr.zb1* suppresses transgene expression in muscle**. The synthetic 3′ untranslated region utr.zb1 reduces expression in both slow and fast muscle, but not in heart or notochord. Epifluorescence from Kaede (green) and TagRFPT (red) in *y323-Gal4, UAS:Kaede, UAS:epNTR-TagRFPT-utr.zb1* **(A,B)** or *y274-Gal4, UAS:Kaede, UAS:epNTR-TagRFPT-utr.zb1* **(C,D)** embryos imaged at 3 dpf. In *y323Et*, Gal4 is expressed outside the brain in slow muscle, notochord, heart and caudal fin. Slow muscle (**A**, asterisk) expression is suppressed by *utr.zb1* but expression in notochord (**B**, bracket), heart (arrowhead), and caudal fin remains intact. Similarly expression of Gal4 in fast muscle is suppressed by *utr.zb1* in *y274Et* (**C**, asterisk), but notochord expression (**D**, bracket) is not diminished.

Supplemental Figure 6 **Annotated sequence of *utr.zb3***.

## References

[B1] Amo R. Aizawa H. Takahoko M. Kobayashi M. Takahashi R. Aoki T.. (2010). Identification of the zebrafish ventral habenula as a homolog of the mammalian lateral habenula. J. Neurosci. 30, 1566–1574. 10.1523/JNEUROSCI.3690-09.2010 20107084 PMC6633804

[B2] Asakawa K. Suster M. L. Mizusawa K. Nagayoshi S. Kotani T. Urasaki A.. (2008). Genetic dissection of neural circuits by Tol2 transposon-mediated Gal4 gene and enhancer trapping in zebrafish. Proc. Natl. Acad. Sci. U.S.A. 105, 1255–1260. 10.1073/pnas.0704963105 18202183 PMC2234125

[B3] Bae Y. K. Kani S. Shimizu T. Tanabe K. Nojima H. Kimura Y.. (2009). Anatomy of zebrafish cerebellum and screen for mutations affecting its development. Dev. Biol. 330, 406–426. 10.1016/j.ydbio.2009.04.013 19371731

[B4] Baek D. Villen J. Shin C. Camargo F. D. Gygi S. P. Bartel D. P. (2008). The impact of microRNAs on protein output. Nature 455, 64–71. 10.1038/nature07242 18668037 PMC2745094

[B5] Bartel D. P. (2009). MicroRNAs: target recognition and regulatory functions. Cell 136, 215–233. 10.1016/j.cell.2009.01.002 19167326 PMC3794896

[B6] Bayer T. A. Campos-Ortega J. A. (1992). A transgene containing lacZ is expressed in primary sensory neurons in zebrafish. Development 115, 421–426. 1425333 10.1242/dev.115.2.421

[B7] Bergeron S. A. Carrier N. Li G. H. Ahn S. Burgess H. A. (2015). Gsx1 expression defines neurons required for prepulse inhibition. Mol. Psychiatry 20, 974–985. 10.1038/mp.2014.106 25224259 PMC4362800

[B8] Bergeron S. A. Hannan M. C. Codore H. Fero K. Li G. Moak Z. B.. (2012). Brain selective transgene expression in zebrafish using an NRSE derived motif. Front. Neural Circuits 6:110. 10.3389/fncir.2012.00110 23293587 PMC3531662

[B9] Bernardos R. L. Raymond P. A. (2006). GFAP transgenic zebrafish. Gene Exp. Patterns 6, 1007–1013. 10.1016/j.modgep.2006.04.006 16765104

[B10] Burgess H. A. Johnson S. L. Granato M. (2009). Unidirectional startle responses and disrupted left-right co-ordination of motor behaviors in robo3 mutant zebrafish. Genes Brain Behav. 8, 500–511. 10.1111/j.1601-183X.2009.00499.x 19496826 PMC2752477

[B11] Choi J. Dong L. Ahn J. Dao D. Hammerschmidt M. Chen J. N. (2007). FoxH1 negatively modulates flk1 gene expression and vascular formation in zebrafish. Dev. Biol. 304, 735–744. 10.1016/j.ydbio.2007.01.023 17306248 PMC1876740

[B12] Davison J. M. Akitake C. M. Goll M. G. Rhee J. M. Gosse N. Baier H.. (2007). Transactivation from Gal4-VP16 transgenic insertions for tissue-specific cell labeling and ablation in zebrafish. Dev. Biol. 304, 811–824. 10.1016/j.ydbio.2007.01.033 17335798 PMC3470427

[B13] Faucherre A. Lopez-Schier H. (2011). Delaying Gal4-driven gene expression in the zebrafish with morpholinos and Gal80. PLoS ONE 6:e16587. 10.1371/journal.pone.0016587 21298067 PMC3027692

[B14] Gohl D. M. Silies M. A. Gao X. J. Bhalerao S. Luongo F. J. Lin C. C.. (2011). A versatile *in vivo* system for directed dissection of gene expression patterns. Nat. Methods 8, 231–237. 10.1038/nmeth.1561 21473015 PMC3079545

[B15] Groth A. C. Olivares E. C. Thyagarajan B. Calos M. P. (2000). A phage integrase directs efficient site-specific integration in human cells. Proc. Natl. Acad. Sci. U.S.A. 97, 5995–6000. 10.1073/pnas.090527097 10801973 PMC18547

[B16] Higashijima S.- I. Okamoto H. Ueno N. Hotta Y. Eguchi G. (1997). High-frequency generation of transgenic zebrafish which reliably express GFP in whole muscles or the whole body by using promoters of zebrafish origin. Dev. Biol. 192, 289–299. 10.1006/dbio.1997.8779 9441668

[B17] Hong E. Santhakumar K. Akitake C. A. Ahn S. J. Thisse C. Thisse B.. (2013). Cholinergic left-right asymmetry in the habenulo-interpeduncular pathway. Proc. Natl. Acad. Sci. U.S.A. 110, 21171–21176. 10.1073/pnas.1319566110 24327734 PMC3876215

[B18] Horstick E. J. Jordan D. C. Bergeron S. A. Tabor K. M. Serpe M. Feldman B.. (2015). Increased functional protein expression using nucleotide sequence features enriched in highly expressed genes in zebrafish. Nucleic Acids Res. 43, e48. 10.1093/nar/gkv035 25628360 PMC4402511

[B19] Hu G. Goll M. G. Fisher S. (2011). PhiC31 integrase mediates efficient cassette exchange in the zebrafish germline. Dev. Dyn. 240, 2101–2107. 10.1002/dvdy.22699 21805532 PMC3938014

[B20] Jenett A. Rubin G. Ngo T.-T. B. Shepherd D. Murphy C. Dionne H.. (2012). A GAL4-driver line resource for Drosophila neurobiology. Cell Rep. 2, 991–1001. 10.1016/j.celrep.2012.09.011 23063364 PMC3515021

[B21] Jessen J. R. Willett C. E. Lin S. (1999). Artificial chromosome transgenesis reveals long-distance negative regulation of rag1 in zebrafish. Nat. Genet. 23, 15–16. 10.1038/12609 10471489

[B22] Jungke P. Hammer J. Hans S. Brand M. (2015). Isolation of Novel CreERT2-driver lines in zebrafish using an unbiased gene trap approach. PLoS ONE 10:e0129072. 10.1371/journal.pone.0129072 26083735 PMC4471347

[B23] Kawakami K. Takeda H. Kawakami N. Kobayashi M. Matsuda N. Mishina M. (2004). A transposon-mediated gene trap approach identifies developmentally regulated genes in zebrafish. Dev. Cell 7, 133–144. 10.1016/j.devcel.2004.06.005 15239961

[B24] Kimura Y. Hisano Y. Kawahara A. Higashijima S. (2014). Efficient generation of knock-in transgenic zebrafish carrying reporter/driver genes by CRISPR/Cas9-mediated genome engineering. Sci. Rep. 4:6545. 10.1038/srep06545 25293390 PMC4189020

[B25] Kimura Y. Satou C. Fujioka S. Shoji W. Umeda K. Ishizuka T.. (2013). Hindbrain V2a neurons in the excitation of spinal locomotor circuits during zebrafish swimming. Curr. Biol. 23, 843–849. 10.1016/j.cub.2013.03.066 23623549

[B26] Kimura Y. Satou C. Higashijima S. (2008). V2a and V2b neurons are generated by the final divisions of pair-producing progenitors in the zebrafish spinal cord. Development 135, 3001–3005. 10.1242/dev.024802 18684740

[B27] Kinkhabwala A. Riley M. Koyama M. Monen J. Satou C. Kimura Y.. (2011). A structural and functional ground plan for neurons in the hindbrain of zebrafish. Proc. Natl. Acad. Sci. U.S.A. 108, 1164–1169. 10.1073/pnas.1012185108 21199947 PMC3024665

[B28] Koga A. Cheah F. S. Hamaguchi S. Yeo G. H. Chong S. S. (2008). Germline transgenesis of zebrafish using the medaka Tol1 transposon system. Dev. Dyn. 237, 2466–2474. 10.1002/dvdy.21688 18729212

[B29] LaFave M. C. Varshney G. K. Burgess S. M. (2015). GeIST: a pipeline for mapping integrated DNA elements. Bioinformatics 31, 3219–3221. 10.1093/bioinformatics/btv350 26049161 PMC4592334

[B30] Lillesaar C. Stigloher C. Tannhauser B. Wullimann M. F. Bally-Cuif L. (2009). Axonal projections originating from raphe serotonergic neurons in the developing and adult zebrafish, Danio rerio, using transgenics to visualize raphe-specific pet1 expression. J. Comp. Neurol. 512, 158–182. 10.1002/cne.21887 19003874

[B31] Masai I. Lele Z. Yamaguchi M. Komori A. Nakata A. Nishiwaki Y.. (2003). N-cadherin mediates retinal lamination, maintenance of forebrain compartments and patterning of retinal neurites. Development 130, 2479–2494. 10.1242/dev.00465 12702661

[B32] McLean D. L. Fan J. Higashijima S. Hale M. E. Fetcho J. R. (2007). A topographic map of recruitment in spinal cord. Nature 446, 71–75. 10.1038/nature05588 17330042

[B33] Mishima Y. Fukao A. Kishimoto T. Sakamoto H. Fujiwara T. Inoue K. (2012). Translational inhibition by deadenylation-independent mechanisms is central to microRNA-mediated silencing in zebrafish. Proc. Natl. Acad. Sci. U.S.A. 109, 1104–1109. 10.1073/pnas.1113350109 22232654 PMC3268308

[B34] Mueller T. Wullimann M. F. (2005). Atlas of Early Zebrafish Brain Development. A Tool for Molecular Neurogenetics. Amsterdam: Elsevier B.V.

[B35] Mueller T. (2012). What is the Thalamus in Zebrafish? Front. Neurosci. 6:64. 10.3389/fnins.2012.00064 22586363 PMC3345571

[B36] Nachtigall P. G. Dias M. C. Pinhal D. (2014). Evolution and genomic organization of muscle microRNAs in fish genomes. BMC Evol. Biol. 14:196. 10.1186/s12862-014-0196-x 25253178 PMC4177693

[B37] Nechiporuk A. Linbo T. Poss K. D. Raible D. W. (2007). Specification of epibranchial placodes in zebrafish. Development 134, 611–623. 10.1242/dev.02749 17215310

[B38] Otsuna H. Hutcheson D. A. Duncan R. N. McPherson A. D. Scoresby A. N. Gaynes B. F.. (2015). High-resolution analysis of central nervous system expression patterns in zebrafish Gal4 enhancer-trap lines. Dev. Dyn. 244, 785–796. 10.1002/dvdy.24260 25694140 PMC4449297

[B39] Parinov S. Kondrichin I. Korzh V. Emelyanov A. (2004). Tol2 transposon-mediated enhancer trap to identify developmentally regulated zebrafish genes *in vivo*. Dev. Dyn. 231, 449–459. 10.1002/dvdy.20157 15366023

[B40] Park H. C. Kim C. H. Bae Y. K. Yeo S. Y. Kim S. H. Hong S. K.. (2000). Analysis of upstream elements in the HuC promoter leads to the establishment of transgenic zebrafish with fluorescent neurons. Dev. Biol. 227, 279–293. 10.1006/dbio.2000.9898 11071755

[B41] Peng H. Chung P. Long F. Qu L. Jenett A. Seeds A. M.. (2011). BrainAligner: 3D registration atlases of Drosophila brains. Nat. Methods 8, 493–500. 10.1038/nmeth.1602 21532582 PMC3104101

[B42] Pfeiffer B. D. Jenett A. Hammonds A. S. Ngo T. T. Misra S. Murphy C.. (2008). Tools for neuroanatomy and neurogenetics in Drosophila. Proc. Natl. Acad. Sci. U.S.A. 105, 9715–9720. 10.1073/pnas.0803697105 18621688 PMC2447866

[B43] Pfeiffer B. D. Ngo T. T. Hibbard K. L. Murphy C. Jenett A. Truman J. W.. (2010). Refinement of tools for targeted gene expression in Drosophila. Genetics 186, 735–755. 10.1534/genetics.110.119917 20697123 PMC2942869

[B44] Pitman J. L. McGill J. J. Keegan K. P. Allada R. (2006). A dynamic role for the mushroom bodies in promoting sleep in Drosophila. Nature 441, 753–756. 10.1038/nature04739 16760979

[B45] Pittman A. J. Law M. Y. Chien C. B. (2008). Pathfinding in a large vertebrate axon tract: isotypic interactions guide retinotectal axons at multiple choice points. Development 135, 2865–2871. 10.1242/dev.025049 18653554 PMC2562560

[B46] Pool A.-H. Kvello P. Mann K. Cheung S. K. Gordon M. D. Wang L.. (2014). Four GABAergic interneurons impose feeding restraint in Drosophila. Neuron 83, 164–177. 10.1016/j.neuron.2014.05.006 24991960 PMC4092013

[B47] Portugues R. Feierstein C. E. Engert F. Orger M. (2014). Whole-brain activity maps reveal stereotyped, distributed networks for visuomotor behavior. Neuron 81, 1328–1343. 10.1016/j.neuron.2014.01.019 24656252 PMC4448760

[B48] Preibisch S. Saalfeld S. Tomancak P. (2009). Globally optimal stitching of tiled 3D microscopic image acquisitions. Bioinformatics 25, 1463–1465. 10.1093/bioinformatics/btp184 19346324 PMC2682522

[B49] Provost E. Rhee J. Leach S. D. (2007). Viral 2A peptides allow expression of multiple proteins from a single ORF in transgenic zebrafish embryos. Genesis 45, 625–629. 10.1002/dvg.20338 17941043

[B50] Randlett O. Wee C. L. Naumann E. A. Nnaemeka O. Schoppik D. Fitzgerald J. E. (2015). Whole-brain activity mapping onto a zebrafish brain atlas. Nat. Methods 12, 1039–1046. 10.1038/nmeth.3581 PMC4710481 26778924

[B51] Rath M. Nitschke R. Filippi A. Ronneberger O. Driever W. (2012). Generation of high quality multi-view confocal 3D datasets of zebrafish larval brains suitable for analysis using Virtual Brain Explorer (ViBE-Z) software. Nat. Methods 9, 735–742. 10.1038/protex.2012.031 22706672

[B52] Rohlfing T. Maurer C. R. Jr. (2003). Nonrigid image registration in shared-memory multiprocessor environments with application to brains, breasts, and bees. IEEE Trans. Inf. Technol. Biomed. 7, 16–25. 10.1109/TITB.2003.808506 12670015

[B53] Ronneberger O. Liu K. Rath M. Ruebeta D. Mueller T. Skibbe H.. (2012). ViBE-Z: a framework for 3D virtual colocalization analysis in zebrafish larval brains. Nat. Methods 9, 735–742. 10.1038/nmeth.2076 22706672

[B54] Rozen S. Skaletsky H. (2000). Primer3 on the WWW for general users and for biologist programmers, in Bioinformatics Methods and Protocols: Methods in Molecular Biology, eds Krawetz S. Misener S. (Totowa, NJ: Humana Press), 365–386. 10.1385/1-59259-192-2:365 10547847

[B55] Satou C. Kimura Y. Higashijima S. (2012). Generation of multiple classes of V0 neurons in zebrafish spinal cord: progenitor heterogeneity and temporal control of neuronal diversity. J. Neurosci. 32, 1771–1783. 10.1523/JNEUROSCI.5500-11.2012 22302816 PMC6703360

[B56] Satou C. Kimura Y. Hirata H. Suster M. L. Kawakami K. Higashijima S. (2013). Transgenic tools to characterize neuronal properties of discrete populations of zebrafish neurons. Development 140, 3927–3931. 10.1242/dev.099531 23946442

[B57] Schindelin J. Arganda-Carreras I. Frise E. Kaynig V. Longair M. Pietzsch T.. (2012). Fiji: an open-source platform for biological-image analysis. Nat. Methods 9, 676–682. 10.1038/nmeth.2019 22743772 PMC3855844

[B58] Schneider C. A. Rasband W. S. Eliceiri K. W. (2012). NIH Image to ImageJ: 25 years of image analysis. Nat. Methods 9, 671–675. 10.1038/nmeth.2089 22930834 PMC5554542

[B59] Scott E. K. Baier H. (2009). The cellular architecture of the larval zebrafish tectum, as revealed by gal4 enhancer trap lines. Front. Neural Circuits 3:13. 10.3389/neuro.04.013.2009 19862330 PMC2763897

[B60] Scott E. K. Mason L. Arrenberg A. B. Ziv L. Gosse N. J. Xiao T.. (2007). Targeting neural circuitry in zebrafish using GAL4 enhancer trapping. Nat. Methods 4, 323–326. 10.1038/nmeth1033 17369834

[B61] Sivasubbu S. Balciunas D. Davidson A. Pickart M. Hermanson S. Wangensteen K.. (2006). Gene-breaking transposon mutagenesis reveals an essential role for histone H2afza in zebrafish larval development. Mech. Dev. 123, 513–529. 10.1016/j.mod.2006.06.002 16859902

[B62] Sprague J. Bayraktaroglu L. Clements D. Conlin T. Fashena D. Frazer K.. (2006). The Zebrafish Information Network: the zebrafish model organism database. Nucleic Acids Res. 34, D581–D585. 10.1093/nar/gkj086 16381936 PMC1347449

[B63] Stockinger P. Kvitsiani D. Rotkopf S. Tirián L. Dickson B. J. (2005). Neural Circuitry that governs Drosophila Male courtship behavior. Cell 121, 795–807. 10.1016/j.cell.2005.04.026 15935765

[B64] Subtelny A. O. Eichhorn S. W. Chen G. R. Sive H. Bartel D. P. (2014). Poly(A)-tail profiling reveals an embryonic switch in translational control. Nature 508, 66–71. 10.1038/nature13007 24476825 PMC4086860

[B65] Suster M. L. Sumiyama K. Kawakami K. (2009). Transposon-mediated BAC transgenesis in zebrafish and mice. BMC Genomics 10:477. 10.1186/1471-2164-10-477 19832998 PMC2768751

[B66] Tabor K. M. Bergeron S. A. Horstick E. J. Jordan D. C. Aho V. Porkka-Heiskanen T. (2014). Direct activation of the Mauthner cell by electric field pulses drives ultra-rapid escape responses. J. Neurophysiol. 112, 834–844. 10.1152/jn.00228.2014 24848468 PMC4122749

[B67] Tay T. L. Ronneberger O. Ryu S. Nitschke R. Driever W. (2011). Comprehensive catecholaminergic projectome analysis reveals single-neuron integration of zebrafish ascending and descending dopaminergic systems. Nat. Commun. 2, 171. 10.1038/ncomms1171 21266970 PMC3105308

[B68] Ulitsky I. Shkumatava A. Jan C. H. Subtelny A. O. Koppstein D. Bell G. W.. (2012). Extensive alternative polyadenylation during zebrafish development. Genome Res. 22, 2054–2066. 10.1101/gr.139733.112 22722342 PMC3460199

[B69] Urasaki A. Morvan G. Kawakami K. (2006). Functional dissection of the Tol2 transposable element identified the minimal cis-sequence and a highly repetitive sequence in the subterminal region essential for transposition. Genetics 174, 639–649. 10.1534/genetics.106.060244 16959904 PMC1602067

[B70] Varshney G. K. Lu J. Gildea D. E. Huang H. Pei W. Yang Z.. (2013). A large-scale zebrafish gene knockout resource for the genome-wide study of gene function. Genome Res. 23, 727–735. 10.1101/gr.151464.112 23382537 PMC3613589

[B71] Wienholds E. Kloosterman W. P. Miska E. Alvarez-Saavedra E. Berezikov E. de Bruijn E.. (2005). MicroRNA expression in zebrafish embryonic development. Science 309, 310–311. 10.1126/science.1114519 15919954

[B72] Xi Y. Yu M. Godoy R. Hatch G. Poitras L. Ekker M. (2011). Transgenic zebrafish expressing green fluorescent protein in dopaminergic neurons of the ventral diencephalon. Dev. Dyn. 240, 2539–2547. 10.1002/dvdy.22742 21932324

[B73] Xie X. Mathias J. R. Smith M. A. Walker S. L. Teng Y. Distel M.. (2012). Silencer-delimited transgenesis: NRSE/RE1 sequences promote neural-specific transgene expression in a NRSF/REST-dependent manner. BMC Biol. 10:93. 10.1186/1741-7007-10-93 23198762 PMC3529185

[B74] Yokogawa T. Hannan M. C. Burgess H. A. (2012). The dorsal raphe modulates sensory responsiveness during arousal in zebrafish. J. Neurosci. 32, 15205–15215. 10.1523/JNEUROSCI.1019-12.2012 23100441 PMC3535275

[B75] Yong W. Otsuna H. Chi-Bin C. Hansen C. (2012). FluoRender: An application of 2D image space methods for 3D and 4D confocal microscopy data visualization in neurobiology research. Pacific Visualization Symposium (PacificVis), 2012 IEEE, 201–208. 10.1109/pacificvis.2012.6183592 PMC3622106 23584131

[B76] Zhang Y. Werling U. Edelmann W. (2012). SLiCE: a novel bacterial cell extract-based DNA cloning method. Nucleic Acids Res. 40, e55. 10.1093/nar/gkr1288 22241772 PMC3333860

