# Supplementary material for: A 3D Searchable Database of Transgenic Zebrafish Gal4 and Cre Lines for Functional Neuroanatomy Studies
Source: Front Neural Circuits. 2015 Nov 24;9:78. doi: 10.3389/fncir.2015.00078 (PMC4656851; doi:10.3389/fncir.2015.00078)
Supplement: Supplemental Table 3 — Summary of recommended CMTK parameters for registration of image stacks acquired using live vglut2a:DsRed fluorescence as the reference channel. [file Table3.DOC]

| **Parameters** | **Recommended** | **Rationale** |
| --- | --- | --- |
| **registrationx** |  |  |
| ***--dofs*** | 12 | improved NCC |
| ***--max-stepsize*** | 32 | minimizes % failed registrations, but increases CPU time |
| ***--min-stepsize*** | 1 | reduces CPU time with negligible impact on NCC |
| ***--stepfactor*** | 0.5 *(default)* |  |
| ***--delta-f-threshold*** | 0 *(default)* |  |
| ***--sampling*** | 1 *(default)* |  |
| ***--coarsest*** | -1 *(default)* |  |
| ***--init*** | --fov *(default)* |  |
| ***--registration-metric*** | --nmi *(default)* |  |
| ***--interpolation*** | --linear *(default)* |  |
|  |  |  |
| **warpx** |  |  |
| ***--grid-spacing*** | 100 | for reference dimensions ~1000 x 600 x 400 pixels |
| ***--grid-refine*** | 2 |  |
| ***--delay-refine*** | no *(default)* |  |
| ***--ignore-edge*** | 0 *(default)* |  |
| ***--no-adaptive-fix*** | no *(default)* |  |
| ***--adaptive-fix-thresh*** | 0.25 | reduces CPU time |
| ***--jacobian-constraint-weight*** | 0 *(default)* |  |
| ***--smoothness-constraint-weight*** | 0.1 | maximizes inter-NCC / intra-NCC |
| ***--inverse-consistency-weight*** | 0 *(default)* |  |
| ***--constraint-relaxation-factor*** | -1 *(default)* |  |
| ***--max-stepsize*** | -1 *(default)* |  |
| ***--min-stepsize*** | 0.25 | reduces CPU time |
| ***--stepfactor*** | 0.5 *(default)* |  |
| ***--delta-f-threshold*** | 0 *(default)* |  |
| ***--no-maxnorm*** | no |  |
| ***--sampling*** | 1 *(default)* |  |
| ***--coarsest*** | -1 *(default)* |  |
| ***--omit-original-data*** | no |  |
| ***--accurate/--fast*** | --fast |  |
| ***--registration-metric*** | --nmi *(default)* |  |
| ***--interpolation*** | --linear *(default)* |  |
|  | | |

**Supplemental Table 3**
