# Supplementary figures and images for: A 3D Searchable Database of Transgenic Zebrafish Gal4 and Cre Lines for Functional Neuroanatomy Studies
Source: Front Neural Circuits. 2015 Nov 24;9:78. doi: 10.3389/fncir.2015.00078 (PMC4656851; doi:10.3389/fncir.2015.00078)

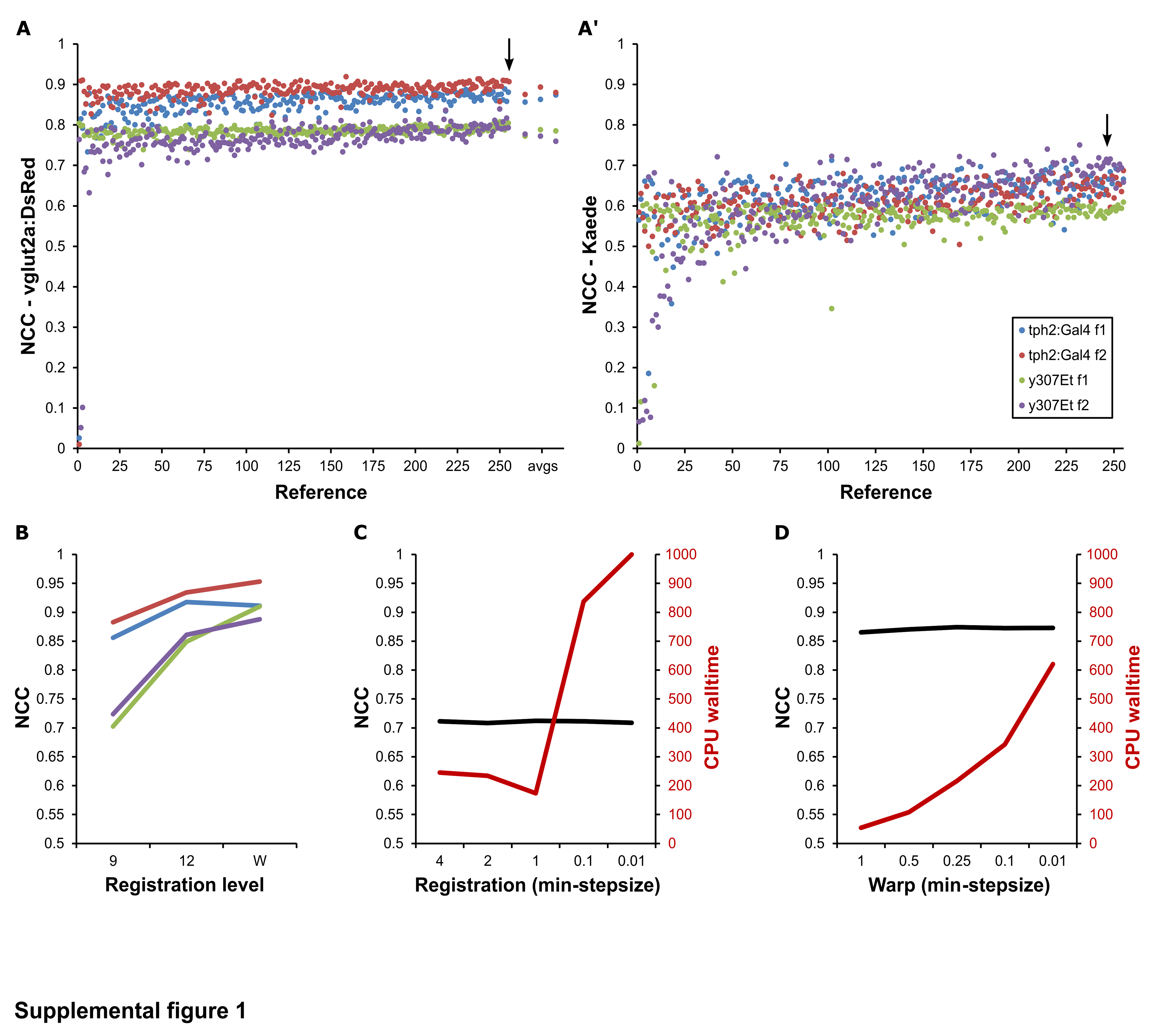

Supplement: Supplemental Figure 1 — Optimization of brain registration using CMTK. (A) Normalized cross-correlation (NCC) for duplicate confocal scans of the vglut2a:DsRed channel in each of four brains in the calibration set (blue: tph:gal4 fish 1; red: tph2:gal4 fish 2; green: y307Et fish 1; purple: y307Et fish 2), after registration by full affine transformation to each of 255 vglut2a:DsRed expressing reference brains. Empty pixels were excluded from the computation using the reference brains as a mask. For each reference, the mean of the four NCCs was taken, and used to provide a rank (higher ranks to the right). In addition, the top 5, 10, and 20 reference brains (based on mean NCC) were used to generate iterative shaped averages (a5, a10, and a20 respectively) whose performance as a reference was also assessed. (A′) NCC for the Gal4/Kaede channel from the same set of calibration and reference brains, however no mask was used before the NCC computation (see Methods). Arrows in (A,A′) indicate the reference brain we selected to use for subsequent registrations. (B) Comparison of the average NCC of the calibration set using 9° of freedom rigid transformation (9: translation, rotation, and anisotropic scale), full affine transformation (12: translation, rotation, anisotropic scale, and sheer), and affine plus warp transformation (W: translation, rotation, anisotropic scale, sheer, and nonrigid). Line colors indicate larvae as in (A).While a number of registration parameters were optimized to maximize the average NCC of the calibration set (see Supplemental Table 3 for full list of tested and recommended parameters), a number of parameters showed no improvement in the average NCC of the calibration set, and so settings that minimized CPU walltime were selected instead. (C) Rigid registrations showed negligible improvement in NCC beyond a min-stepsize of 1 while greatly increasing the CPU time. Thus, a min-stepsize setting of 1 was used for registrations. (D) Nonrigid registrations showed negligible i [file Image1.TIF]

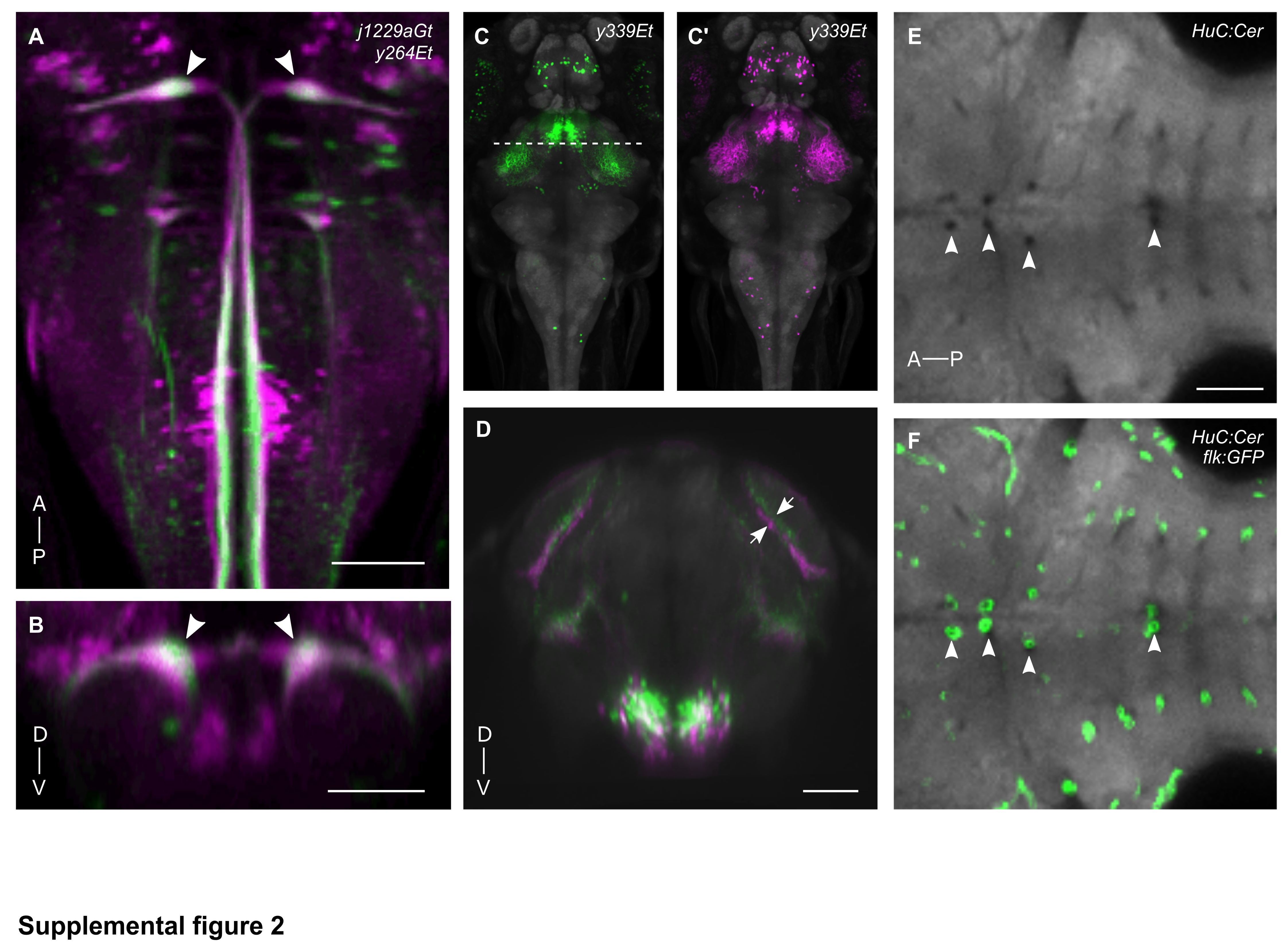

Supplement: Supplemental Figure 2 — Assessing registration accuracy by co-localized Gal4 reporter gene expression. Dorsal (A) and transverse (B) views of brain browser projections for enhancer trap line y264Tg (green) and transgenic line j1229aGt (magenta) in the region including the Mauthner cells (arrowheads). (C,C′) Dorsal view of maximum projection of average representations of y339Et, made from two independent sets of three embryos each. Dotted line shows location of transverse slice shown in (D). (D) Slice through the midbrain and diencephalon of superimposed y339Et averages. In the right optic tectum, a neuropil layer (arrows) in the averages is misaligned by 1–2 cell diameters. (E) Horizontal view of a slice through the hindbrain from HuC:Cer average brain. Arrowheads indicate gaps in the tissue lacking neuronal Cer expression. flk:GFP expression, which labels blood vessels, is superimposed in (F). Anterior (A), Posterior (P), Dorsal (D), Ventral (V). All scale bars 50 μm. [file Image2.TIF]

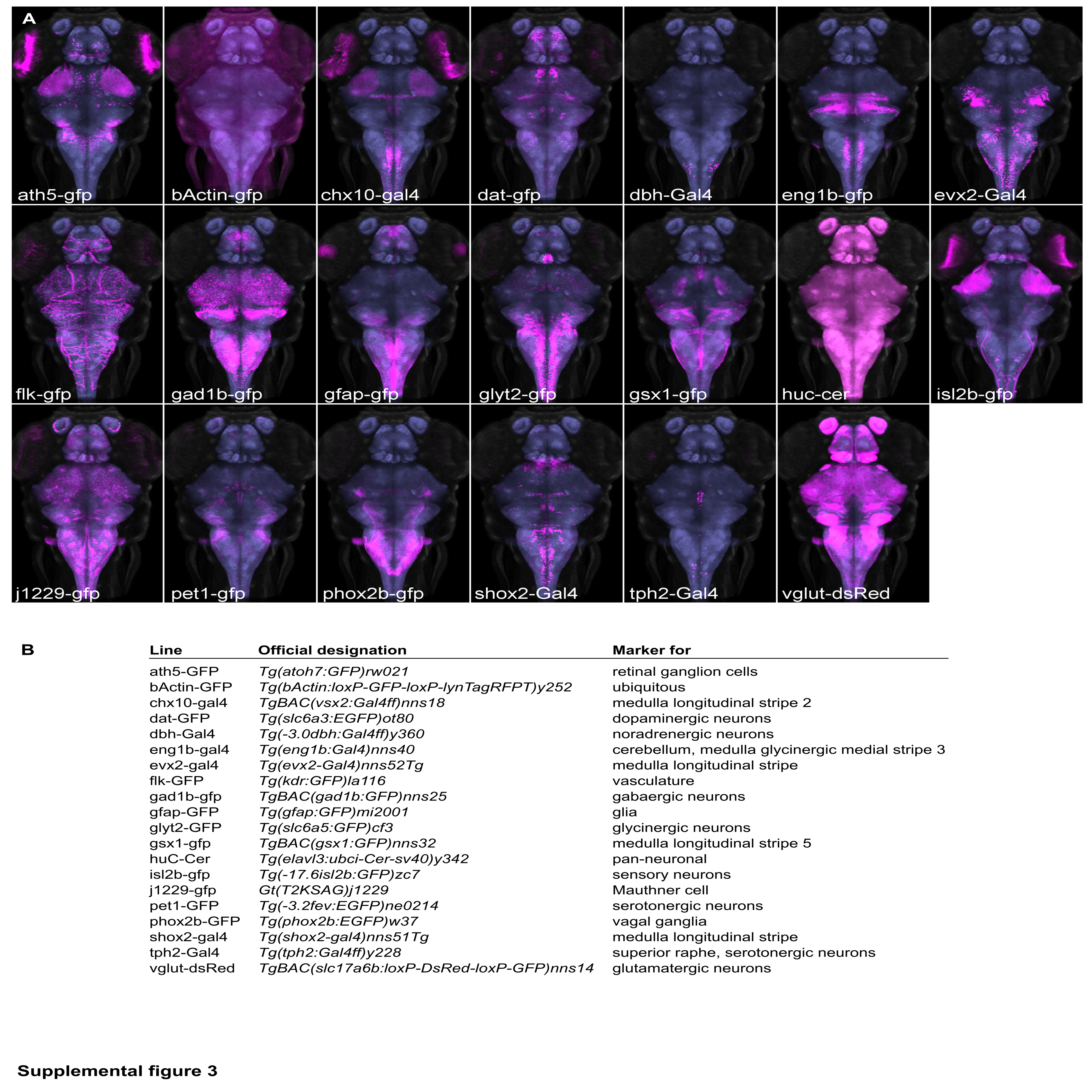

Supplement: Supplemental Figure 3 — Transgenic lines providing neuroanatomical markers. Maximum projections (A) and expression domains (B) of transgenic lines that were imaged as part of this study. Gal4 lines were visualized using UAS:GFP (magenta). Background: ubiquitous βactin:Switch (gray) and pan-neuronal HuC:Cer (blue, after brain mask applied). [file Image3.TIF]

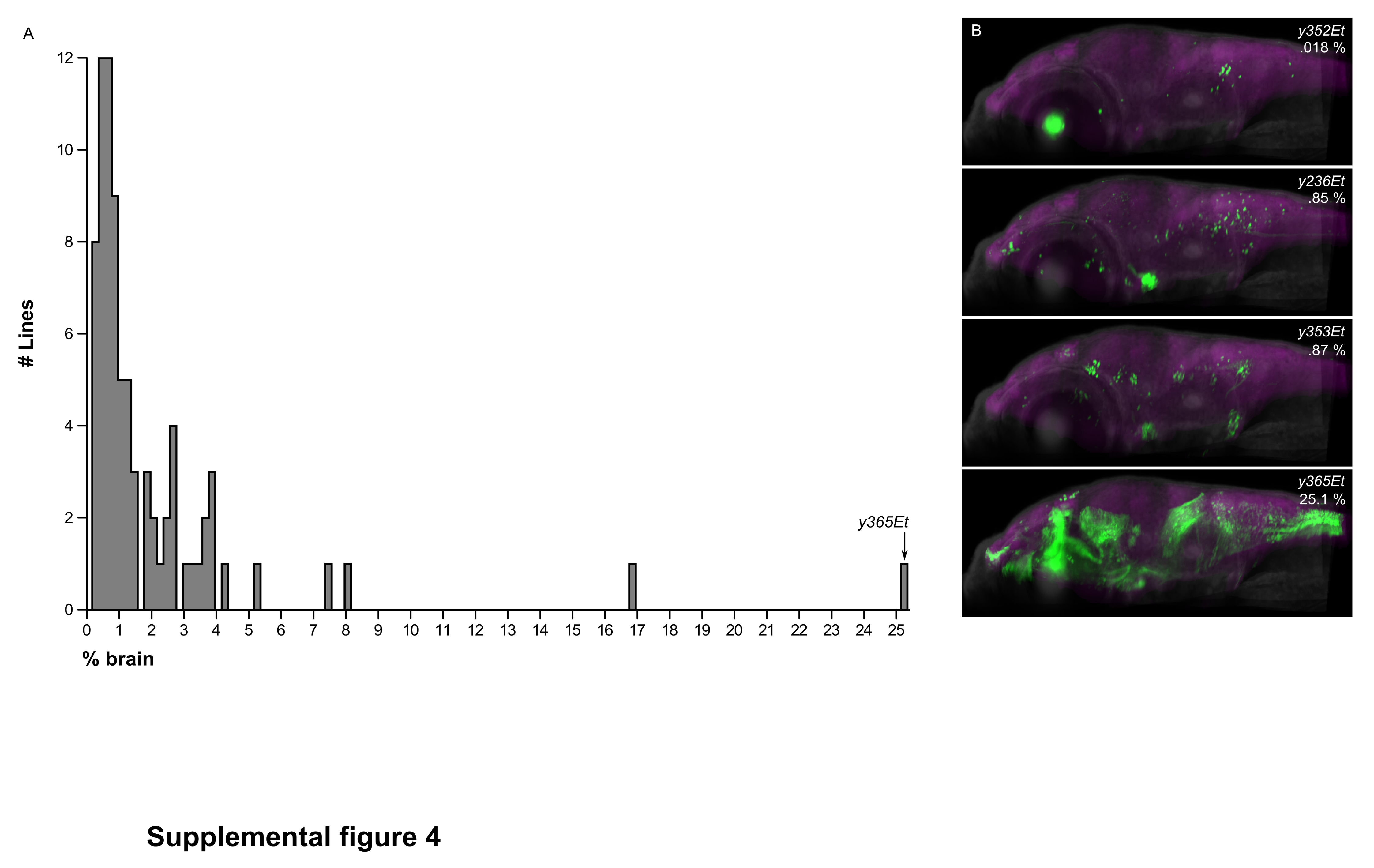

Supplement: Supplemental Figure 4 — Extent of brain expression in Gal4 enhancer trap lines. (A) Histogram of the extent of brain coverage for the 80 Gal4 enhancer trap lines imaged in this study. (B) Sagittal hemi-projection for the line with the most restricted Gal4 expression pattern y352Et, for two lines with close to the median extent of Gal4 expression (y236Et and y353Et) and for the line with the broadest Gal4 expression pattern included in the database, y365Et. [file Image4.TIF]

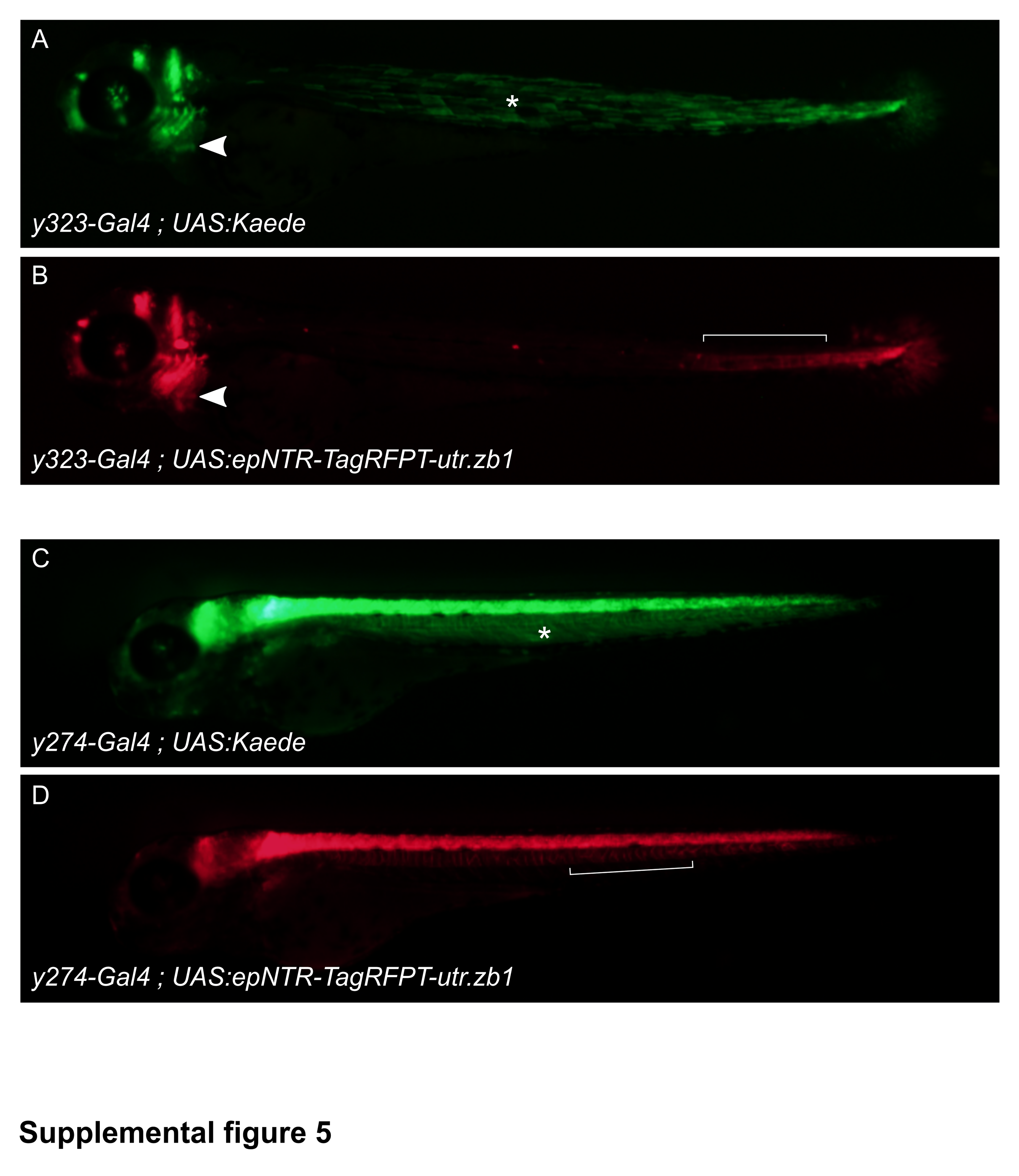

Supplement: Supplemental Figure 5 — Synthetic 3′ UTR utr.zb1 suppresses transgene expression in muscle. The synthetic 3′ untranslated region utr.zb1 reduces expression in both slow and fast muscle, but not in heart or notochord. Epifluorescence from Kaede (green) and TagRFPT (red) in y323-Gal4, UAS:Kaede, UAS:epNTR-TagRFPT-utr.zb1 (A,B) or y274-Gal4, UAS:Kaede, UAS:epNTR-TagRFPT-utr.zb1 (C,D) embryos imaged at 3 dpf. In y323Et, Gal4 is expressed outside the brain in slow muscle, notochord, heart and caudal fin. Slow muscle (A, asterisk) expression is suppressed by utr.zb1 but expression in notochord (B, bracket), heart (arrowhead), and caudal fin remains intact. Similarly expression of Gal4 in fast muscle is suppressed by utr.zb1 in y274Et (C, asterisk), but notochord expression (D, bracket) is not diminished. [file Image5.TIF]
