# Supplementary material for: A 3D Searchable Database of Transgenic Zebrafish Gal4 and Cre Lines for Functional Neuroanatomy Studies
Source: Front Neural Circuits. 2015 Nov 24;9:78. doi: 10.3389/fncir.2015.00078 (PMC4656851; doi:10.3389/fncir.2015.00078)
Supplement: Supplemental Figure 6 — Annotated sequence of utr.zb3. [file Image6.PDF]

attaagtctcagccaccgttGAGCAGGTGTTCCATACATTCCATTaaGATCTATTTCAGTTCATTTCAAGT
CAATTAATTTCAGCTCAAGTTTTTCCATTTCATTTCATTTCAAATAAATTACATTCACTTAATTTCAGATC
ATTTCAAATTggatcTTTAGCaCTGTAAAAGTCTTAAATTTCACTCAGATTGGTCTTAAAATGGTCTTAAGAAGTCTT
AAGTTGATCTCAATTCTATTATGGCGCAGACAGACATACATTCCATTATcCTATTAcTAACATTCCAGAGAG
TATCAGAATTTCACGGTACGATAATACCTCGATATGAATGGCACGGTACGCaACACATGAAGACCCACACT
GGAGGGAAACTATTCACATGCACTCAGTGTGGAAAGAGTTTCAACTGATCAGCAAACCTTAATGAACACAT
AAAGATCCACACTGGTGTGAAAGAGTATATGTGCTTTTAGTGTGAGAAGACTTTTATTACACCTCCAAATT
cAAAACCACACCAGACGATTCACACTGGAGAGACCGTACAAGTGTTCAAACaGTGACAAGAGGTTTAATCA
GTTAACACATCTGAAAACACAAAAGAGGATTCACACTGGAcAagcTCAGAATcTCACGGTACGATcAaTCC
aCGcTATGAATcGCACGGTACGCaactgaacatgtcaaaacctgtggagactgttgagatttgatgttctg
aaaagataaagcctataaataaaatgttgcccaaatttcctgcctgatgtttttctttgtctttgctacat
ggctttgctgctcggatcggctcactctgtgtatgccacgttcactttgtactctccttctcacggtaggt
ttattatttttagatgtgcagttagtttctgtgaaataacacaccacacactgatattgtctgtgcattga
cttggtgagtgcacattgtttttgatcttgacatatttatatttgattgatcaggtgaactgtgtgaatct
aaagtgctccatacagatgttctgcattgaaaatattctcattttattagtggaagtgagtgtatgcca

Key

Grey lower case: Ocean pout antifreeze protein 3' untranslated region

Black/highlighted region: microRNA targets from zebrafish genes
- Match to full 8mer seed sequence
- Match to 7mer-1a
- Target regions for:

dre-mir-1-3p
dre-mir-203a-3p
dre-mir-599-5p
dre-mir-126-3p
dre-mir-199-5p
dre-mir-126-3p

Supplemental figure 6
